# Deterioration of Cortical and Trabecular Microstructure Identifies Women With Osteopenia or Normal Bone Mineral Density at Imminent and Long‐Term Risk for Fragility Fracture: A Prospective Study

**DOI:** 10.1002/jbmr.3924

**Published:** 2019-12-10

**Authors:** Roland Chapurlat, Minh Bui, Elisabeth Sornay‐Rendu, Roger Zebaze, Pierre D. Delmas, Danny Liew, Eric Lespessailles, Ego Seeman

**Affiliations:** ^1^ INSERM UMR1033 and Université de Lyon Lyon France; ^2^ Centre for Epidemiology and Biostatistics Melbourne School of Population and Global Health, University of Melbourne Melbourne Australia; ^3^ Department of Medicine and Endocrinology Austin Health, University of Melbourne Melbourne Australia; ^4^ StraxCorp Melbourne Australia; ^5^ School of Public Health and Preventive Medicine Monash University Melbourne Australia; ^6^ IPROS, CHR Orléans, Université d'Orléans Orléans France; ^7^ Mary MacKillop Institute of Healthy Aging, Australian Catholic University Melbourne Australia

**Keywords:** IMMINENT FRACTURE RISK, MICROSTRUCTURAL DETERIORATION, NORMAL BMD, OSTEOPENIA, OSTEOPOROSIS

## Abstract

More than 70% of women sustaining fractures have osteopenia or “normal” bone mineral density (BMD). These women remain undetected using the BMD threshold of −2.5 SD for osteoporosis. As microstructural deterioration increases bone fragility disproportionate to the bone loss producing osteopenia/normal BMD, we hypothesized that the structural fragility score (SFS) of ≥70 units, a measure capturing severe cortical and trabecular deterioration, will identify these women. Distal radial images were acquired using high‐resolution peripheral quantitative tomography in postmenopausal French women, mean age 67 years (range 42–96 years); 1539 women were followed for 4 years (QUALYOR) and 561 women followed for 8 years (OFELY). Women with osteopenia or normal BMD accounted for ~80% of fractures. Women ≥70 years, 29.2% of the cohort, accounted for 39.2% to 61.5% of fractures depending on follow‐up duration. Women having fractures had a higher SFS, lower BMD, and a higher fracture risk assessment score (FRAX) than women remaining fracture‐free. In each BMD category (osteoporosis, osteopenia, normal BMD), fracture incidence was two to three times higher in women with SFS ≥70 than <70. In multivariable analyses, associations with fractures remained for BMD and SFS, not FRAX. BMD was no longer, or weakly, associated with fractures after accounting for SFS, whereas SFS remained associated with fracture after accounting for BMD. SFS detected two‐to threefold more women having fractures than BMD or FRAX. SFS in women with osteopenia/normal BMD conferred an odds ratio for fracture of 2.69 to 5.19 for women of any age and 4.98 to 12.2 for women ≥70 years. Receiver‐operator curve (ROC) analyses showed a significant area under the curve (AUC) for SFS, but not BMD or FRAX for the women ≥70 years of age. Targeting women aged ≥70 years with osteopenia indicated that treating 25% using SFS to allocate treatment conferred a cost‐effectiveness ratio < USD $21,000/QALY saved. Quantifying microstructural deterioration complements BMD by identifying women without osteoporosis at imminent and longer‐term fracture risk. © 2019 The Authors. *Journal of Bone and Mineral Research* published by American Society for Bone and Mineral Research.

## Introduction

The morbidity, mortality, and cost of fragility fractures is increasing, in part, because longevity increases the proportion of the population over 65 years of age.^1^ Indeed, fractures among women and men ≥70 years account for ~70% of direct health care costs and fractures among those with osteopenia account for 50% of costs of treatment.^2^ Two strategies are used to prevent fractures. The most common is to measure bone mineral density (BMD) and target treatment to women with osteoporosis defined as a BMD *T*‐score of −2.5 standardized deviations (SD) or lower.^3^ The second is to identify women with risk factors using the fracture risk assessment (FRAX) score,^4^ especially women at risk for major fragility fractures (of the hip, clinical spine, humerus, and forearm) because they account for ~70% of the morbidity, mortality, and cost of all fractures in the community and predispose to further fractures within 12 months.^5^

Just as the morbidity, mortality, and cost of cerebrovascular disease arises among persons with moderate hypertension,^6^ the burden of fractures arises among the vast majority of postmenopausal women with moderate deficits in BMD designated as “osteopenia” (*T*‐score between −2.5 and −1.0 SD) or so‐called “normal” BMD (*T*‐score above −1 SD), not the smaller numbers of women in the community with severe deficits in BMD designated as “osteoporosis.”^7^, ^8^, ^9^, ^10^, ^11^, ^12^ Among this large postmenopausal population with osteopenia or normal BMD are women at “high” or “imminent” risk for fracture (within 1 to 2 years) needing prompt treatment, and women at intermediate term risk for fractures.^5^, ^13^, ^14^

Curbing the population burden of fractures requires a means of identifying women with bone fragility erroneously perceived to be at low risk because they have osteopenia or normal BMD. A rational approach to meet this challenge is to measure bone microstructural deterioration because deterioration in cortical and trabecular architecture increases bone fragility exponentially, disproportionate to the bone loss producing it and the modest deficits in BMD found in postmenopausal women with osteopenia or so‐called “normal” BMD.^15^, ^16^, ^17^, ^18^

Cross‐sectional studies demonstrate that a measurement of microstructural deterioration distinguishes women with osteopenia and prevalent fractures from women with osteopenia without fractures.^9^, ^19^ Recent prospective studies support the notion that microstructural deterioration identifies women with osteopenia having incident fractures.^18^, ^20^, ^21^

We developed and validated a surrogate of bone fragility relatively free of microstructural determinants of bone strength assembled during growth.^19^ This structural fragility score (SFS) quantifies concurrent cortical and trabecular deterioration relative to their mean peak values in premenopausal women. We tested whether women with osteopenia or normal BMD of any age, but particularly women ≥70 years, at imminent and longer‐term risk for fracture could be identified before they have a fracture by measuring the SFS. We tested whether the SFS did so independent of BMD or FRAX, thereby enhancing the ability to target treatment to women needing it and avoid treating those at low risk. We also modeled the cost‐effectiveness of applying the SFS to select women older than 70 years with osteopenia for treatment.

## Materials and Methods

### Participants

We studied two population‐based cohorts. The OFELY cohort (Os des Femmes de Lyon) is a prospective study of 1039 women started in February 1992.^22^ We focused on the 589 postmenopausal women, aged 68 ± 9 years, with a baseline measurement of bone microstructure obtained during 2006–2008, followed for a median [interquartile range] of 9.4 [1.0] years. The QUALYOR cohort was composed of 1539 women followed for 5 years; 1042 were recruited in Lyon and 497 in Orléans, France, based on having *T*‐scores at the hip or spine between −1.0 and −2.5 SD with clinical risk factors for fracture or −3.0 SD without clinical risk factor.^22^, ^23^ There were no differences in the proportions of women with osteoporosis in the OFELY and QUALYOR cohorts (6.7% and 7.8%, respectively, *p* = 0.37) or with osteopenia or normal BMD (93.3% in OFELY and 92.2% in QUALYOR). Missing values for BMD, FRAX, or SFS resulted in exclusion of 28 women, leaving 2100 women. Consent was obtained from all participants. These studies were approved by the CPP Sud‐Est II institutional review boards, Lyon, France.

### Measurements

Vertebral and nonvertebral fractures were confirmed using radiographs, the dual x‐ray absorptiometry vertebral fracture assessment or reports. Fractures of the head, toes, and fingers were excluded. Femoral neck BMD was measured using Hologic Discovery A in QUALYOR and QDR 4500 in OFELY. *T*‐scores were calculated using NHANES III. Distal radial images were acquired using HRpQCT (Xtreme CT, Scanco Medical AG, Brüttisellen, Switzerland).^24^ Radiation exposure is under 3 microsievert. Quality control was monitored by daily scans of hydroxyapatite rods (QRM, Moehrendorf, Germany). Cortical and trabecular microstructure were quantified using StrAx1.0 (StraxCorp, Melbourne, Australia).^25^

### The structural fragility score

**Figure 1:**
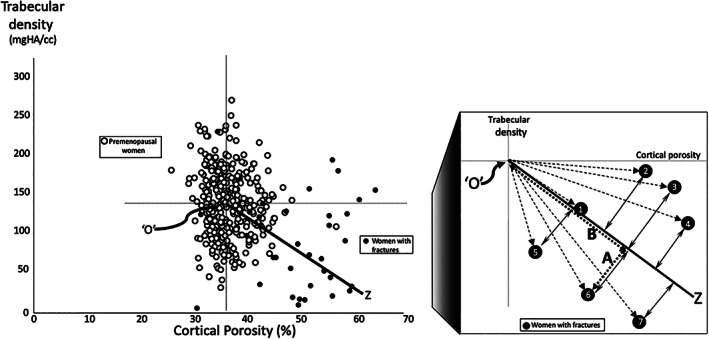
As adapted from Zebaze and colleagues,^19^ trabecular density is plotted as a function of cortical porosity (left). Point O is the mean of each trait in healthy premenopausal women (open circles). Black circles denote postmenopausal women with fragility fractures. The black regression line O to Z captures the concurrent deterioration in cortical porosity and trabecular density. (Right) The derivation of the structural fragility score (SFS). Black circles 1 to 7 represent postmenopausal women with fragility fractures. Slope of line O to Z is the mean of all the slopes of lines from O to each woman with fractures (hatched lines). In the example for patient 6, the distance B (dotted line) captures the concurrent deterioration in both traits in absolute terms relative to O, and the distance A (dotted line) captures the deterioration in one trait relative to the other. For patients 2, 3, and 4, the deterioration in cortical porosity is greater relative to deterioration in trabecular density. For patients 5, 6, and 7, the deterioration in trabecular density is greater relative to the deterioration in cortical porosity. The SFS captures these varying absolute and relative deteriorations as explained in Materials and Methods.

We hypothesized that a surrogate measure of bone fragility will capture both the absolute and relative deterioration in cortical and trabecular bone produced by age‐ and menopause‐related bone loss. Demonstration of deterioration in both cortical and trabecular bone is needed because bone loss affects both traits. A deficit in only one trait is likely to reflect errors in positioning of the region of interest, not microstructural deterioration.^19^ Figure 1 is simplified to more clearly explain the derivation of the SFS. O is the mean of cortical porosity and trabecular density in 324 healthy premenopausal women. The slope of the regression line was derived using regression analysis of these traits in 33 postmenopausal women with fractures.^19^ A woman's (*x*, *y*) values are projected onto the regression line to quantify the absolute and relative deterioration in these two traits. For women with (*x*, *y*) coordinates on the regression line, distance B is the absolute deterioration in cortical and trabecular bone. The further the (*x*, *y*) coordinates are from O along the regression line, the greater the absolute deterioration in both traits and the greater the distance B. The perpendicular distance A captures the differing relative deficits. Women with coordinates above the regression line have relatively more severe cortical than trabecular deterioration. Women with (*x*, *y*) values below the regression line have the opposite. The SFS = B – A. For women with (*x*, *y*) coordinates on the regression line, A is zero. When distance A is large, the greater the likelihood that there is a deficit in only one trait, suggesting the deficit is the result of an error in positioning the region of interest, not bone loss. The precision of the SFS (acquisition, repositioning, and co‐registration) in 15 women having three measurements was 1.12% expressed as the root mean square of the coefficient of variation.

### Analyses

De‐identified data were analyzed at the University of Melbourne. The thresholds used were a BMD *T*‐score ≤ − 2.5 SD and FRAX (with BMD) score > 20. The SFS threshold of 70 was derived by the Youden method, which maximizes the sum of the sensitivity and specificity.^26^ The threshold was established in a different cohort^19^ and was confirmed here, being 70.01 for women having fractures of any type and major fragility fractures during 2 years, 67.4 and 69.7, respectively, during 4 years, and 70.33 and 72.34, respectively, during 8 years. Summary statistics for all data and separately for the two cohorts are presented as mean and standard deviation (SD) for normally distributed data and two‐sample *t* tests were used to compare fracture and nonfracture groups. For non‐normally distributed data, median and interquartile range (IQR) were used as summary statistics and the nonparametric Mann–Whitney test was used to compare groups. For binary variables, summary statistics are presented by cell frequency and percentage. Exact logistic regression was used to compute odds ratios (OR) and their confidence intervals (CI) for associations between each binary predictor (BMD, FRAX, SFS) and fracture outcome for each cohort. Testing for equality in ORs between two cohorts was conducted using Mantel–Haenszel test, for all data and for subgroups of women of any age with osteopenia or normal BMD, osteoporosis, and women ≥70 years with osteopenia or normal BMD.

In the presence of equality in ORs between two cohorts, analyses were conducted for the pooled cohorts using exact logistic regression for univariate analysis and the penalized maximum likelihood logistic regression for multivariable analysis. Pairwise comparisons between proportion of fracture captured by BMD, FRAX, and SFS were carried out using two‐sample *t* test for proportions, and Bonferroni correction method was used to adjust for multiple testing. For the subgroups, exact logistic regression was used to study association between fracture, FRAX, and SFS. Sensitivity and specificity were also given for these two predictors in the subgroup analysis. The performance of each predictor was assessed using the area under the curve (AUC) for all data and subgroups.

We also analyzed total vBMD, trabecular density, and cortical porosity for women of any age and women aged ≥70 years. The threshold of 231 mgHA/cc was chosen for total vBMD based on the SFS value of 70 units from the fitted regression of SFS on total vBMD. For trabecular density and cortical porosity, the nominal thresholds were the 5th centile (4.8 mgHA/cc) and 90th centile (42.2%), respectively, in premenopausal women. Exact logistic regression or penalized maximum likelihood logistic regression were used to examine associations of these predictors with fractures. All analyses were conducted using STATA (StataCorp, College Station, TX, USA), version 15.0 (http://www.stata.com). A *p* < 0.05 (two‐tailed) denoted statistical significance.

A modeled health economic evaluation was done to compare the outcomes and costs of using the SFS to target women ≥70 years with osteopenia versus current standard care. A decision‐analytic Markov model^27^, ^28^ with 1‐year cycles and three health states (“alive pre‐fracture,” “alive post‐fracture,” and “dead”) was developed to simulate the onset of fragility fractures and death. The incremental capacity of SFS to detect women at risk of fractures was as reported in the findings of this article. The cost of SFS was assumed to be USD $210 per person using HR‐pQCT. Data were obtained from published sources regarding the risks of fractures (5.8% per year),^2^ acute costs of fractures (USD $20,000),^2^ health‐related quality‐of‐life (utility) measures (0.80 among survivors of fractures),^29^ and efficacy of prophylactic therapy (relative fracture risk reduction of 50%).^30^ The costs of therapy were based on government‐subsidized costs via the Australian Pharmaceutical Benefits Scheme,^31^ with weighted‐average costs amounting to USD $510 (AUD $750) per year. The model compared outcomes between use of SFS and current standard care in terms of the number of fractures, years of life lived, quality‐adjusted life years (QALYs) lived, and costs over 5‐year and 10‐year time horizons. A 5% annual discount rate was applied to future health benefits and costs, in line with Australian guidelines.^32^ For the economic evaluation, the output of interest was the incremental cost‐effectiveness ratio (ICERs) in terms of net costs per quality‐adjusted life‐years (QALYs) saved and per year of life saved.

## Results

### Cohort characteristics

At baseline, the women in the OFELY cohort were older than women in the QUALYOR cohort (68.0 versus 65.9 years, *p* < 0.001), and they had a higher SFS (58.5 versus 56.7, *p* = 0.015), FRAX score (8.03 versus 6.36, *p* < 0.001) but higher BMD *T*‐score (−1.36 versus −1.70, *p* < 0.001). However, there were no differences in the proportions of women with osteoporosis in the respective cohorts (OFELY 6.7%, QUALYOR 7.8%, *p* = 0.37) or with osteopenia or normal BMD (OFELY 93.3%, QUALYOR 92.2%). Table 1 shows that in both cohorts, women having incident fractures had a higher baseline SFS, lower BMD, and higher FRAX score than women remaining fracture free. Supplemental Tables S1 and S2 show characteristics of all women and women of each cohort categorized according to their SFS, BMD, and FRAX thresholds. Supplemental Table S3 shows the odds ratios for having any type of fracture or major fragility fractures. Fracture did not differ by cohort or subgroups of each cohort so further analyses are of the pooled cohorts.

**Table 1:** Summary Statistics for SFS, BMD, and FRAX by Fracture Status for the Two Cohorts

		QUALYOR							OFELY						
		Fracture			Nonfracture				Fracture			Nonfracture			
Follow‐up	Variable	*n*	Mean	SD	*n*	Mean	SD	*p* Value	*n*	Mean	SD	*n*	Mean	SD	*p* Value
Any type of fracture															
2 years	SFS	66	62.2	14.9	1473	56.5	13.3	**0.001**	31	67.2	15.7	530	57.9	16.60	**0.003**
	BMD	66	−1.75	0.60	1473	−1.70	0.53	0.415	31	−1.69	0.90	530	−1.34	0.81	**0.019**
	FRAX^a^	66	5.55	3.40	1473	5.30	3.80	0.432	31	8.00	8.70	530	5.50	6.00	**0.001**
4 years	SFS	126	60.9	14.6	1413	56.4	13.2	**<0.001**	57	67.4	16.5	504	57.46	16.41	**<0.001**
	BMD	126	−1.80	0.55	1413	−1.69	0.53	**0.026**	57	−1.73	0.81	504	−1.32	0.81	**<0.001**
	FRAX^a^	126	5.80	4.20	1413	5.20	3.70	0.094	57	9.60	7.30	504	5.30	5.65	**<0.001**
8 years	SFS								106	64.0	16.6	455	57.18	16.44	**<0.001**
	BMD								106	−1.62	0.78	455	−1.30	0.82	**<0.001**
	FRAX^a^								106	8.00	7.70	455	5.20	5.50	**<0.001**
Major fragility fractures															
2 years	SFS	31	64.0	15.6	1508	56.6	13.3	**0.002**	22	68.7	15.9	539	58.0	16.6	**0.003**
	BMD	31	−1.93	0.62	1508	−1.69	0.53	**0.014**	22	−1.70	0.93	539	−1.34	0.82	**0.045**
	FRAX^a^	31	5.90	3.60	1508	5.30	3.75	0.053	22	9.50	7.00	539	5.50	6.00	**0.047**
4 years	SFS	61	63.0	14.7	1478	56.5	13.2	**<0.001**	37	70.9	15.6	524	57.6	16.4	**<0.001**
	BMD	61	−1.93	0.56	1478	−1.69	0.53	**0.001**	37	−1.79	0.87	524	−1.33	0.81	**0.001**
	FRAX^a^	61	6.00	4.20	1478	5.30	3.70	**0.004**	37	12.0	9.80	524	5.40	5.90	**0.001**
8 years	SFS								65	67.3	16.0	496	57.3	16.4	**<0.001**
	BMD								65	−1.69	0.81	496	−1.32	0.81	**0.001**
	FRAX^a^								65	8.40	8.10	496	5.30	5.75	**<0.001**

SFS = structural fragility score; FRAX = fracture risk assessment; BMD = bone mineral density.

a Summary statistics for FRAX are presented as median and interquartile (IQR).

### The fracture burden

Table 2 shows the proportion of women having fractured during 2, 4, and 8 years was higher in women with osteoporosis than women with osteopenia/normal BMD. However, the greater numbers of women with osteopenia/normal BMD made this BMD category the source of ~80% of the fracture burden. Women aged ≥70 years were also an important source of the fracture burden. They comprised 29.2% of the cohort but accounted for 39.2% (38/97), 39.9% (73/183), and 51.9% (55/106) of women having fractures of any type, and 45.3% (24/53), 49% (48/98), and 61.5% (40/65) of women having major fragility fractures during 2, 4, and 8 years, respectively. Table 2 also shows that, of the women with osteopenia/normal BMD, only a small percentage, ranging from 1.96% to 23.1%, had fractures during 2 to 8 years of follow‐up.

**Table 2:** Duration of Follow‐Up Since Baseline Assessment of Bone Microstructure Is Shown for Women of Any Age and Women ≥70 Years Having Any Type of Fracture or Major Fragility Fractures

Follow‐up	Any type of fracture			Major fragility fractures		
(years)	All	Osteoporosis	Osteopenia/normal BMD	All	Osteoporosis	Osteopenia /normal BMD
	*n/N* (%)	*n/N* (%) (incidence)	*n/N* (%) (incidence)	n (%)	*n/N* (%) (incidence)	*n/N* (%) (incidence)
Women of any age						
2	97/2100 (4.6%)	17/114 (17.5%) (14.9%)	80/1986 (82.5%) (4.03%)	53/2100 (2.5%)	14/114 (26.4%) (12.3%)	39/1986 (73.6%) (1.96%)
4	183/2100 (8.7%)	26/114 (14.2%) (22.8%)	157/1986 (85.8%) (7.91%)	98/2100 (4.7%)	22/114 (22.4%) (19.3%)	76/1986 (77.6%) (3.83%)
8	106/561 (18.9%)	14/38 (13.2%) (36.9%)	92/523 (86.8% (17.6%)	65/561 (11.6%)	10/38 (15.4%) (26.3%)	55/523 (84.6%) (10.5%)
Women 70 years and older						
2	38/613 (6.2%)	10/47 (26.3%) (21.3%)	28/566 (73.7%) (4.95%)	24/613 (3.9%)	7/47 (29.2%) (14.9%)	17/566 (70.8%) (3.00%)
4	73/613 (11.9%)	16/47 (21.9%) (34.0%)	57/566 (78.1%) (10.1%)	48/613 (7.8%)	12/47 (25.0%) (25.5%)	36/566 (75.0%) (6.36%)
8	55/214 (25.7%)	12/28 (21.8%) (42.9%)	43/186 (78.2%) (23.1%)	40/214 (18.7%)	9/28 (22.5%) (32.1%)	31/186 (77.5%) (16.7%)

*n/N* denotes number of women having a fracture per total number of women followed; also expressed as a percentage (%). Also shown is the *n/N* of women with osteoporosis or osteopenia/normal bone mineral density (BMD) having fractures as a percent of all women in the corresponding BMD category.

*N* = sample size in all data or subgroups; *n* = number of fractures in all data or subgroups; % = percentage of fracture contributed by subgroup; incidence = percentage of fracture in all data or subgroups.

### Detecting women having incident fractures

**Figure 2:**
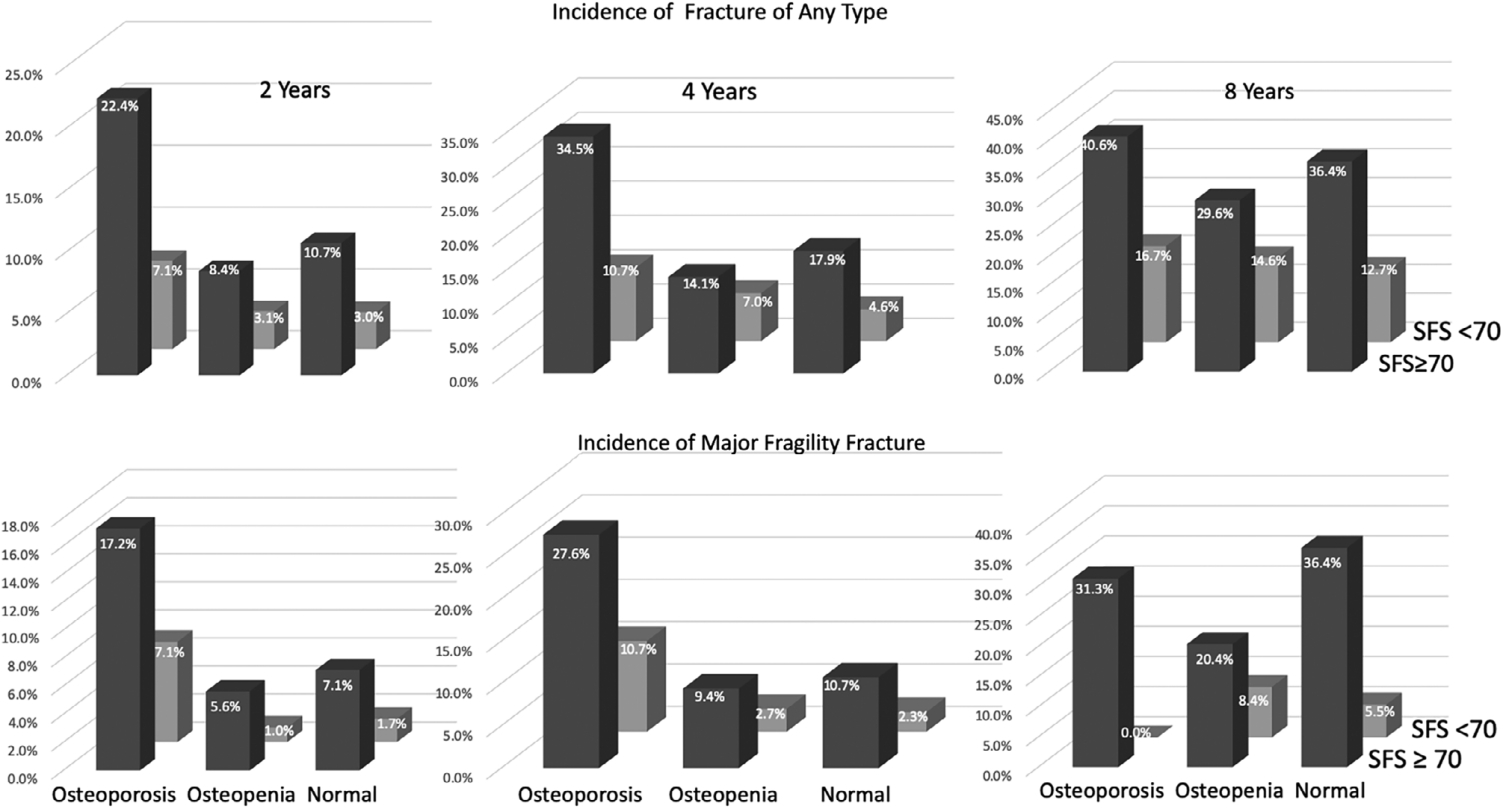
The incidence of women of all ages having a fragility fracture of any type or major fragility fractures during 2, 4, and 8 years stratified by category of bone mineral density (BMD) and structural fragility score (SFS) above the threshold of 70 (dark gray columns) or below it (light gray columns).

We assessed the ability of SFS, BMD, and FRAX thresholds to detect these small percentages of women having incident fragility fractures among the majority remaining fracture free. Figure 2 shows that in each category of BMD, two to three times more women with SFS ≥70 had a fracture than women with SFS <70.

**Figure 3:**
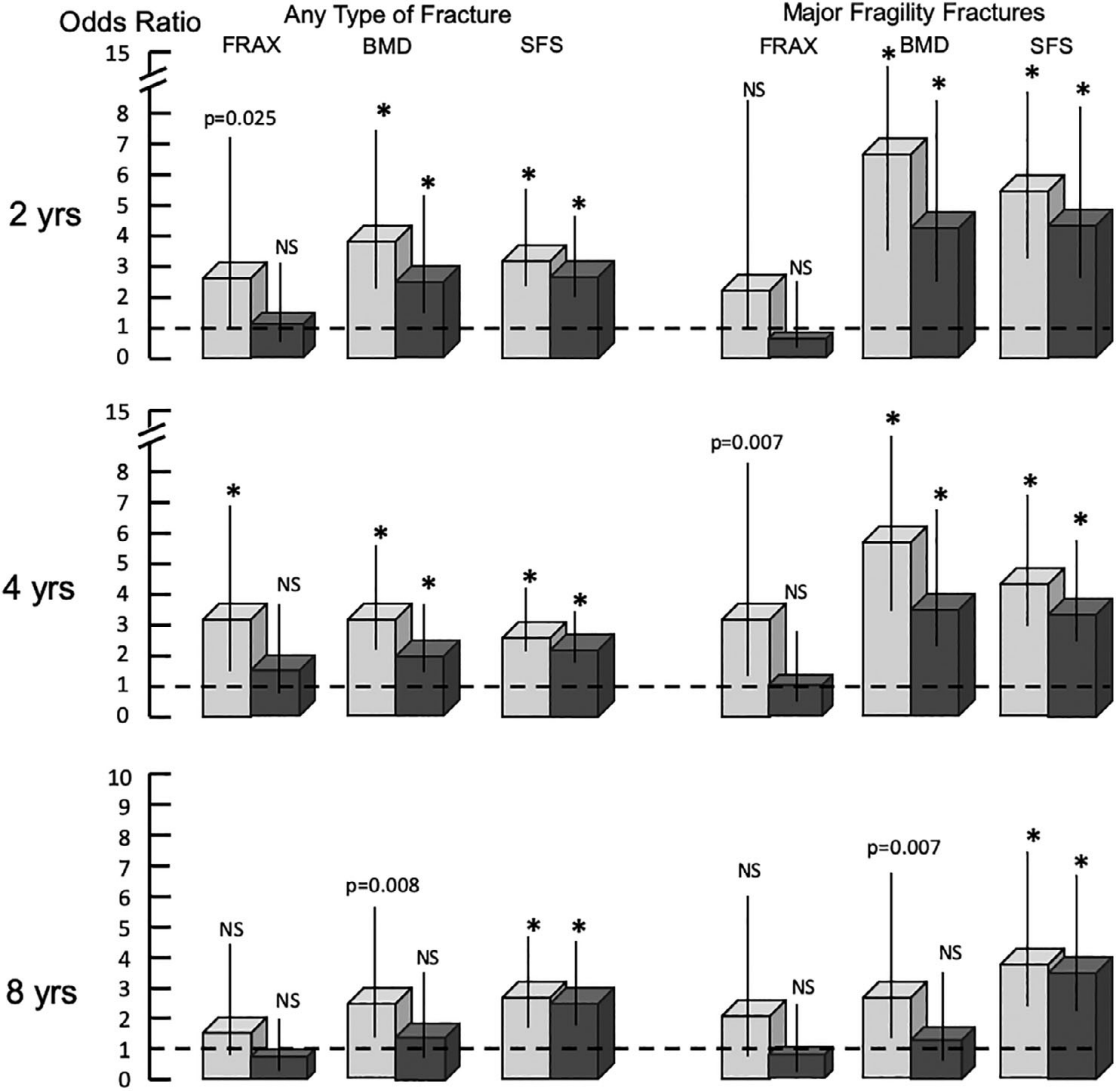
Odds ratios for fracture risk assessment (FRAX) score, bone mineral density (BMD), and the structural fragility score (SFS) before (light gray) and after (dark gray) adjusting for other predictors in multivariate analyses. *p ≤ 0.0001.

**Figure 4:**
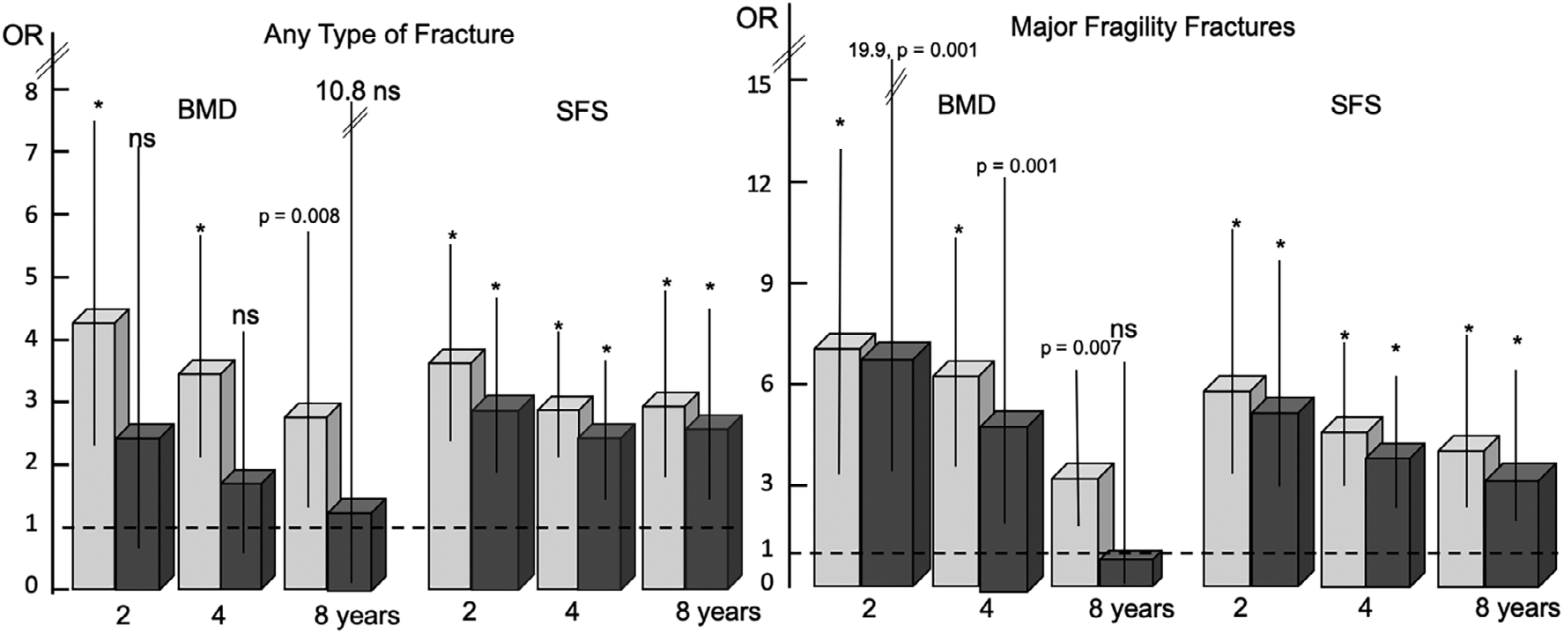
The light gray bars (95% confidence intervals) show the odds ratios (ORs) associated with bone mineral density (BMD) and the structural fragility score (SFS) and incident fractures of any type and major fragility fractures in the univariate analysis. The dark gray bars show the ORs between BMD and fractures are no longer significant after accounting for the SFS but remain significant between the SFS and fractures after accounting for BMD. *p < 0.0001.

Figure 3 shows BMD and SFS, not FRAX, were consistently associated with fractures in univariate analyses and that associations with fractures diminished for BMD but remained significant for both BMD and SFS in multivariable analyses. Figure 4 shows BMD was no longer associated with fractures of any type and less strongly associated with major fragility fractures at 4 and 8 years' follow‐up after accounting for SFS, whereas SFS remained associated with fractures after accounting for BMD. Results in Fig. 4 are shown in Supplemental Table S4, which uses a referent of low fracture risk, namely the composite of SFS < 70 (denoting minimal microstructural deterioration) and BMD > − 2.5 SD (denoting no osteoporosis). In the absence of severe microstructural deterioration (SFS < 70), the presence of low BMD (≤−2.5 SD) was not associated with fracture. However, despite the absence of osteoporosis (BMD > −2.5 SD), osteopenia/normal BMD was associated with fracture in the presence of microstructural deterioration (SFS ≥70). The highest OR occurred when SFS ≥70 and BMD ≤−2.5 SD. These observations were found at 2 and 4 years' follow‐up for any type of fracture and major fragility fractures (except at 8 years for the latter due to small numbers of subject size in this category, *n* = 65/562, 11.6%).

Likewise, in a stratification analysis, during 2 years' follow‐up, SFS ≥70 was associated with fractures in women with osteoporosis (OR = 3.76, *p* = 0.029) and in women osteopenia/normal BMD (OR = 3.0, *p* < 0.001). However, BMD ≤−2.5 SD was only associated with fracture in women with SFS ≥70 (ie, microstructural deterioration) (OR = 3.06, *p* = 0.002), not low SFS < 70 (OR = 2.44, *p* = 0.097). The reduction in OR after adjustment was 31.6% for BMD and 13.3% for SFS. The results were similar at 4 years' follow‐up; SFS ≥70 was associated with fracture in women with osteoporosis (OR = 4.39; *p* = 0.004) and osteopenia/normal BMD (OR = 2.40, *p* < 0.001). BMD ≤ −2.5 SD was only associated with fracture in women with SFS ≥70 (OR = 3.14, *p* < 0.001), not SFS < 70 (OR = 1.72, *p* = 0.223). The reduction in OR after adjustment was 25.6% for BMD and 10% for SFS.

**Figure 5:**
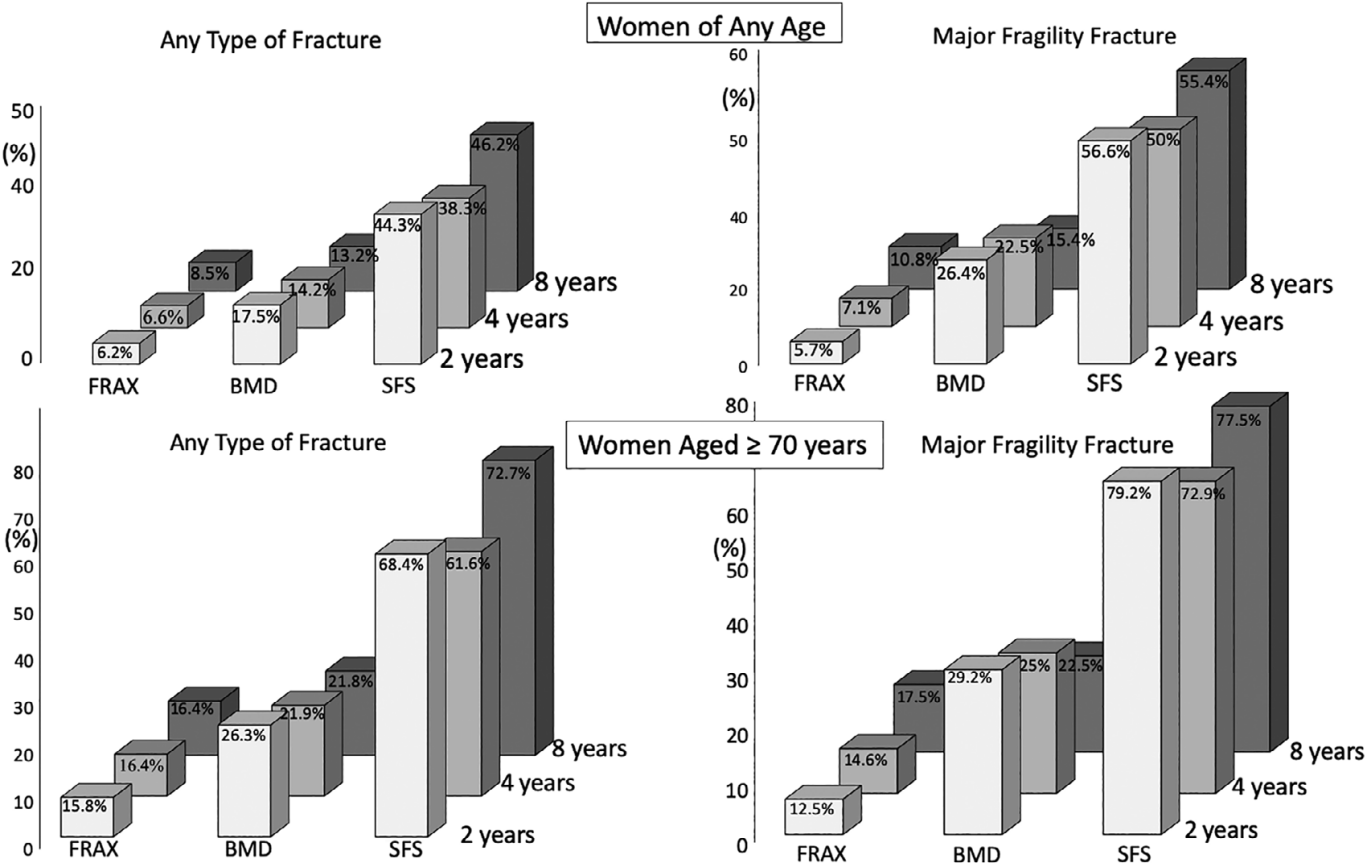
The percent of women of any age (upper two panels) and women ≥70 years (lower two panels) having any type of fracture (left panels) or major fragility fractures (right panels) during 2, 4, and 8 years' follow‐up identified using the thresholds for FRAX, BMD, and SFS. The SFS detects two‐ to threefold more women having incident fractures than FRAX or BMD. SFS = structural fragility score; FRAX = fracture risk assessment score; BMD = bone mineral density. See Supplemental Table S5 for details, Supplemental Table S6 for comparisons of the three tools for the whole data set, and Supplemental Table S7.

Figure 5 shows the SFS detected ~38% to 56% of women of any age and ~60% to 80% of women ≥70 years of age having any type or major fragility fractures, several‐fold more women than detected using BMD or FRAX during 2, 4 and 8 years' follow‐up. (See Supplemental Tables S1, S2, and S5 for details of sample sizes above and below the thresholds for these tools.) Supplemental Table S6 compares the tools; SFS was significantly better than FRAX and BMD, whereas BMD was significantly better than FRAX but not in women ≥70 years of age.

**Figure 6:**
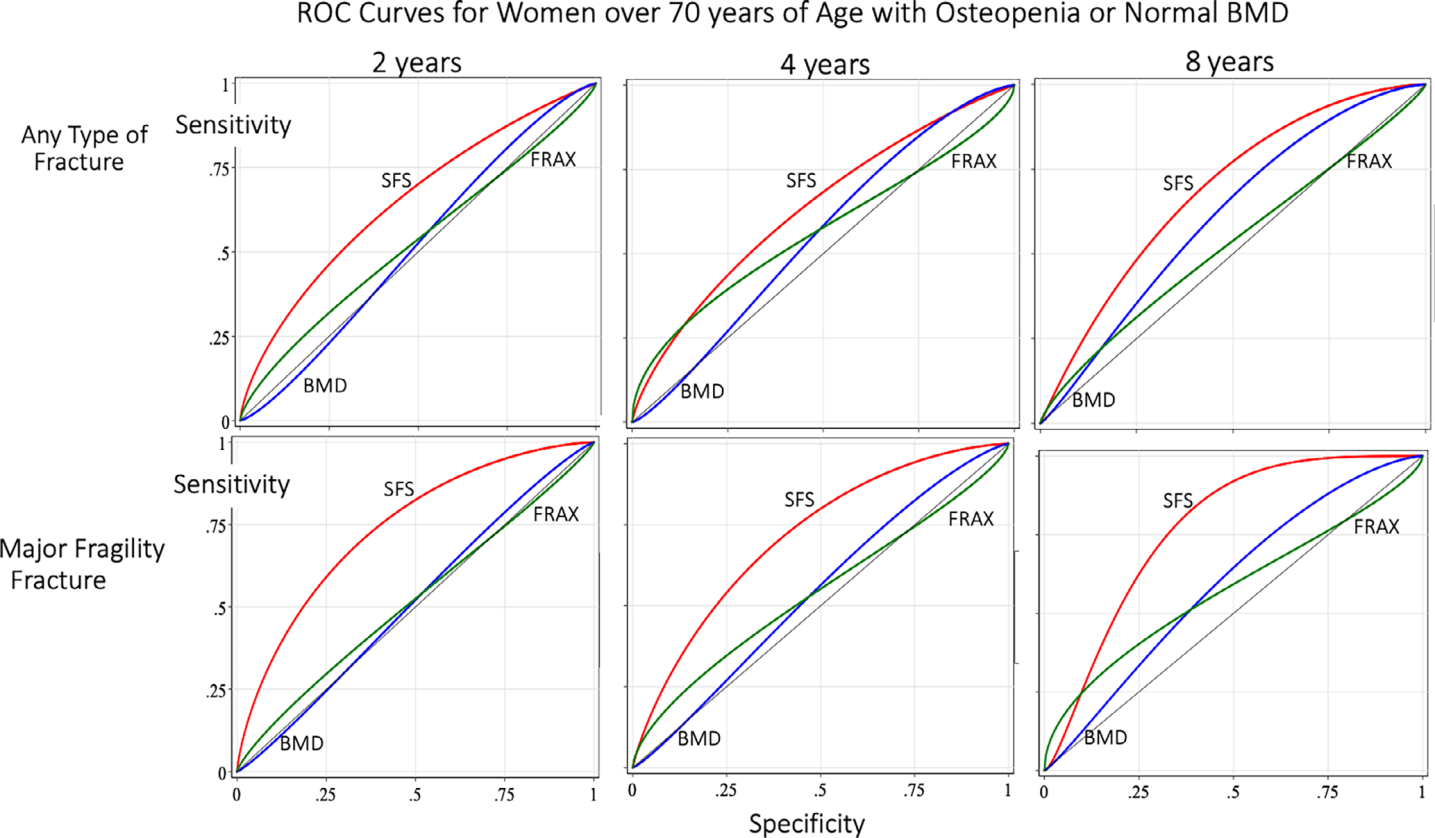
Area under the receiver‐operator curves (ROC) at 2, 4, and 8 years for women ≥70 years of age with osteopenia showing greater area under the curve for the structural fragility score (SFS) than the fracture risk assessment (FRAX) score or bone mineral density (BMD). See Supplemental Tables S8 and S9.

Table 3 shows the odds ratios, sensitivity, and specificity of FRAX and SFS in women with osteopenia/normal BMD and women with osteoporosis. Supplemental Table S7 shows the sample sizes according to thresholds. FRAX was associated with fractures of any type at 4 years only (*p* = 0.034). SFS was associated with fractures of any type and major fragility fractures at 2, 4, and 8 years in women with osteopenia/normal BMD conferring odds between 2.69 to 5.19 for women of any age and 4.98 to 12.2 for women ≥70 years, and corresponding sensitivities and specificities as shown in Table 3. Associations with fractures were less consistent in the small numbers of women with osteoporosis (*n* = 114 but only 38 followed for 8 years). Figure 6 shows the ROC curves with significant AUC for SFS, not BMD or FRAX, for the women ≥70 years of age. Details of AUC (95% CI) for each tool are shown in Supplemental Table S8 (any type of fracture) and Supplemental Table S9 (major fragility fracture).

**Table 3:** The Odds Ratio (OR) With 95% Confidence Intervals (CI), Sensitivity, Specificity, and *p* Value for Women of Any Age and Women ≥70 Years With Osteopenia or Normal BMD and Women With Osteoporosis, Having Fractures of Any Type or a Major Fragility Fracture During 2, 4, and 8 Years

Follow‐up		Fracture of any type				Major fragility fractures		
(years)	OR (95% CI)	Sensitivity	Specificity	*p* Value	OR (95% CI)	Sensitivity	Specificity	*p* Value
Women of any age with osteopenia or normal BMD								
2 FRAX	1.55 (0.18; 6.28)	2.5%	98.4%	0.387	1.57 (0.04; 9.96)	2.56%	98.4%	0.483
SFS	3.00 (1.81; 4.89)	37.5%	83.3%	**<0.0001**	5.19 (2.60; 10.4)	51.3%	83.2%	**<0.0001**
4 FRAX	2.65 (0.88; 6.69)	3.82%	98.5%	**0.041**	2.57 (0.49; 8.57)	3.95%	98.4%	0.130
SFS	2.40 (1.64; 3.47)	31.8%	83.7%	**<0.0001**	3.89 (2.35; 6.37)	43.4%	83.5%	**<0.0001**
8 FRAX	1.59 (0.36; 5.39)	4.35%	97.2%	0.500	2.97 (0.67; 10.3)	7.27%	97.4%	0.076
SFS	2.70 (1.61; 4.47)	39.1%	80.7%	**<0.0001**	3.62(1.94;6.68)	47.3%	80.1%	**<0.0001**
Women 70 years and over with osteopenia or normal BMD								
2 FRAX	1.40 (0.15; 6.09)	7.14%	94.8%	0.654	1.12 (0.03; 7.72)	5.88%	94.7%	0.609
SFS	4.79 (2.03; 11.9)	64.3%	72.7%	**0.0001**	12.2 (3.35; 67.3)	82.3%	72.5%	**<0.0001**
4 FRAX	2.37 (0.76; 6.33)	10.5%	95.3%	0.108	1.69 (0.31; 5.95)	8.33%	94.9%	0.428
SFS	3.34 (1.84; 6.07)	54.4%	73.7%	**<0.0001**	6.33 (2.90; 14.6)	69.4%	73.6%	**<0.0001**
8 FRAX	1.36 (0.30; 5.06)	9.30%	93.0%	0.741	2.13 (0.46; 812)	12.9%	93.6%	0.257
SFS	4.06 (1.87; 8.98)	65.1%	68.5%	**0.0001**	4.98 (2.01; 13.1)	71.0%	67.1%	**0.0001**
Women of any age with osteoporosis								
2 FRAX	1.97 (0.41; 7.81)	23.5%	86.6%	0.280	0.94 (0.09; 4.94)	14.3%	85.0%	0.999
SFS	3.71 (1.05; 16.8)	76.5%	53.6%	**0.034**	2.69 (0.71; 12.5)	71.4%	52.0%	0.153
4 FRAX	2.10 (0.56; 7.10)	23.1%	87.5%	0.213	1.35 (0.29; 5.07)	18.2%	85.9%	0.739
SFS	4.39 (1.49; 14.5)	76.9%	56.8%	**0.003**	3.14 (1.05; 10.7)	72.7%	54.3%	**0.032**
8 FRAX	0.78 (0.16; 3.64)	35.7%	58.3%	0.999	0.58 (0.08; 3.24)	30.0%	57.1%	0.746
SFS	3.33 (0.32; 174)	92.9%	20.8%	0.383	3.42 (0.43, NA)	100%	21.4%	0.168

SFS = structural fragility score; FRAX = fracture risk assessment; BMD = bone mineral density.

Odds ratio and its confidence interval and *p* value were computed using exact logistic regression.

As shown in Supplemental Table S10, SFS consistently outperformed total vBMD. In particular, in women ≥70 years of age, the ORs for fracture were ~twofold higher than the ORs associated with total vBMD (*p* ranging 0.007 to 0.028). The correlation between SFS and vBMD was −0.83. The threshold of total vBMD corresponding to SFS 70 was 231 mgHA/cc. For women of any age (Supplemental Table S11) and women ≥70 years (Supplemental Table S12), reduced trabecular density was associated with fracture but not in the absence of increased cortical porosity. Likewise, increased cortical porosity was associated with fracture but not in the absence of reduced trabecular density. The association with fracture was present when deficits in trabecular density and increased cortical porosity coexisted as captured by the SFS and conferred ORs for fracture ranging from 2.61 to 5.8 (all *p* < 0.0001).

### Health economics

The modeled economic evaluation indicated that if 25% of the screened population were targeted for treatment using the SFS, this approach was likely to be cost‐effective compared with standard care, with an ICER of USD $19,000 per QALY saved and USD $57,000 per year of life saved over a 5‐year time horizon. Over a 10‐year time horizon, the ICERs were USD $4000 per QALY saved and USD $8000 per year of life saved. Cost‐effectiveness also improved if SFS was to be targeted at a population at higher underlying risk of fracture. If 30% of the screened population were targeted for treatment, the ICER would reduce to USD $15,000 per QALY saved over a 5‐year time horizon and USD $47,000 per year of life saved.

## Discussion

This study provides data supporting the hypothesis that including a measurement of microstructural deterioration complements the use of BMD by identifying women without osteoporosis at imminent, intermediate, and long‐term risk for fragility fracture. Including a measurement of microstructural deterioration detected ~40% to 60% of women of any age and ~60% to 80% of women ≥70 years of age having any type or major fragility fractures, several‐fold more women than detected using BMD or FRAX during 2, 4, and 8 years' follow‐up. Women with osteopenia or normal BMD would otherwise remain undetected using the diagnostic BMD threshold of –2.5 SD alone.

We report that the population burden of fragility fractures arises from two sources. Women with osteopenia or normal BMD accounted for ~80% of all women at imminent (2‐year), intermediate (4‐year), and longer‐term (8‐year) risk for a fragility fracture, not women with osteoporosis. This has been reported in several cross‐sectional studies^7^, ^8^, ^9^, ^10^, ^11^, ^12^ and recently in a large prospective study.^21^ Women over 70 years of age were also an important source of the fracture burden. They comprised ~30% of this cohort but contributed 45% to 60% of all major fragility fractures depending on the duration of follow‐up. Women over 70 years of age are the source of more than 70% of health care costs^2^ and, despite their advanced age, most sustaining incident fractures have osteopenia or so‐called normal BMD, not osteoporosis.

Distinguishing postmenopausal women with osteopenia or normal BMD having fragility fractures from the majority remaining fracture‐free is a formidable challenge because they comprise only a small percentage of all women with osteopenia or normal BMD in the community. By definition, the BMD *T*‐score threshold of –2.5 SD for “osteoporosis” identified none of these women and so only targeting treatment to women with osteoporosis defined as BMD ≤−2.5 SD is unlikely to address the public health burden of fractures. Nor was the FRAX threshold of 20%, a measure of 10‐year risk, sensitive.

The challenge was met, in part, by measuring microstructural deterioration. The BMD measurement does not capture microstructural deterioration. This is a limitation because microstructual deterioration increases fragility disproportionate to the bone loss, causing it and the modest BMD deficits found in postmenopausal women with osteopenia or so‐called “normal” BMD.^15^, ^16^, ^17^ The increase in porosity of a “compact” structure like cortical bone reduces its bending strength to the seventh power. Bone loss of an already porous structure like trabecular or “spongy” bone reduces trabecular bending strength to the third power as trabecular plates thin, perforate, and become disconnected rods.^15^

Within each BMD category (osteoporosis, osteopenia, normal BMD), women with cortical and trabecular microstructural deterioration captured by the SFS ≥70 had a two‐ to threefold higher fracture incidence than women with a SFS < 70. From Table 3, measuring SFS conferred respective sensitivities/specificities for women ≥70 years with osteopenia/normal BMD, having fractures of any type of 64.3%/72.7% (2 years), 54.4/73.7% (4 years), and 65.1/68.5%, (8 years) and having major fragility fractures 82.3/72.5% (2 years), 69.4/73.6% (4 years) and 71%/67.1% (8 years).

The desired property of a surrogate of bone fragility is that it captures the microstructural basis of that fragility. Cortical “porosity” is widely regarded as being the result of bone loss. However, absolute values of cortical porosity (and trabecular density) are the net result of their growth‐dependent assembly, which confers bone strength, and age‐ and menopause‐related bone loss, which confers bone fragility. More than 80% of “pores” are cross sections of Haversian canals in the center of osteons formed during growth.^19^, ^33^, ^34^, ^35^, ^36^ Osteons with their central fluid‐filled canal, the circumferential lamellae of differently orientated mineralized collagen fibers, and the cement line separating osteons from each other and from interosteonal (interstitial) bone obstruct or deflect microcrack propagation, while trabecular plates connect with each other and buttress the cortices conferring bone strength, not fragility.^37^ The SFS serves as a tool to identify women at high risk for fracture because it is relatively free of the morphological determinants of bone strength. It expresses the age‐related deterioration in cortical porosity and trabecular density relative to their respective peak mean values in healthy premenopausal women, not their absolute values, which are weakly predictive of prevalent or incident fractures as reported recently,^20^, ^21^ and confirmed in this study (Supplemental Tables S10 and S11).

The SFS was also designed to capture concurrent cortical and trabecular deterioration. The presence of coexisting deficits makes it likely that these deficits are the result of bone loss because bone loss is global. A deficit in only one compartment is likely to reflect positioning of the region of interest (ROI).^19^, ^38^ It is intriguing that adjacent cross sections of bone are assembled using similar volumes of bone matrix.^38^, ^39^ The differing external dimensions and internal microstructure are assembled using differing void volumes, not differing matrix volumes. Distally, the large size of the rhomboidal‐shaped radial metaphysis is assembled using more void volume, not more matrix volume. Most of this constant matrix volume is used to form the thin porous cortex and high trabecular density, so a distally positioned ROI suggests cortical bone loss but no trabecular bone loss. Proximally, the narrow tubular metaphyseal‐diaphyseal region is fashioned using less void volume, not less matrix volume. Here, most of this constant matrix volume is used to form a thick, compact cortical shell of low porosity with little, if any, trabecular bone within the narrow medullary canal. A proximally positioned ROI suggests no cortical bone loss but trabecular bone loss; hence, the need to measure both compartments and compare them to a control at precisely the same location. Errors in these requirements may partly explain the modest or poorly predictive value of cortical porosity or trabecular density alone but greater predictive strength of combined cortical and trabecular deficits as reported previously^19^ and in this study.

In a cross‐sectional study,^19^ BMD was no longer associated with prevalent fracture after accounting for the SFS, whereas the association between SFS and prevalent fracture remained after accounting for BMD. Moreover, coexisting deficits in cortical and trabecular bone, not isolated deficits, were associated with prevalent fracture in that study. Likewise, in this prospective study, BMD was no longer associated with the incidence of any type of fracture after accounting for SFS and more weakly associated with the occurrence of major fragility fractures at 4 and 8 years' follow‐up, whereas the association between SFS and incident fractures of any type or major fragility fractures remained after accounting for BMD (Fig. 4).

Bone loss reduces the amount of bone (captured by BMD) and deteriorates the microstructure of the reduced bone mass (captured by SFS). Both BMD and SFS are associated with fracture; the risk increases as BMD decreases and as SFS increases. Although BMD and SFS were both associated with fracture in univariate analyses, when BMD and SFS were adjusted for each other and for FRAX in multivariable analyses, the ORs for BMD decreased (Fig. 3). In a stratified analysis, SFS was associated with fracture in women with osteoporosis and osteopenia/normal BMD, but BMD was only associated with fracture in women with high SFS (microstructural deterioration). We explored this further by comparing the OR conferred by high SFS and low BMD in the absence of the other predictor. Relative to individuals at low risk with SFS < 70 and BMD > −2.5 SD as a referent, SFS ≥70 alone in women with osteopenia or normal BMD was associated with fractures, but low BMD (ie, osteoporosis) was not associated with fracture in the absence of microstructural deterioration (Fig. 4, Supplemental Table S4).

Finding similar ORs conferred by BMD and SFS but a greater reduction from unadjusted to adjusted ORs for BMD than for SFS is consistent with confounding. The association between alcohol use and lung cancer decreases after accounting for smoking, but the association between smoking and lung cancer remains after accounting for alcohol use. It is the smoking that confers the risk of cancer. We propose that although bone loss reduces bone mass, it is the microstructural deterioration of that reduced amount of bone that confers the risk of fracture because increased cortical porosity and decreased trabecular density reduce bone strength disproportionate to the bone loss producing this deterioration whether BMD is in the osteoporosis, osteopenic, or normal range (Fig. 2).

SFS was a more sensitive predictor of fracture than total vBMD, particularly in women ≥70 years (Supplemental Table S9). A measurement of total vBMD, like a measurement of cortical porosity and trabecular density, is the net result of its accrual during growth and its deterioration during aging. We suggest the SFS outperformed total vBMD, porosity, and trabecular density because it captures their concurrent deterioration during advancing age, relatively free of their accrual during growth.

Bone densitometry has been used to estimate fracture risk for more than 50 years and led to the use of three diagnostic categories: “osteoporosis,” “osteopenia,” and “normal” BMD.^3^, ^40^, ^41^, ^42^ Several misconceptions have arisen using this categorical approach to a continuous variable.

First, although fracture risk increases as BMD decreases, there is unintended dichotomization of fracture risk. Treatment decisions are often mistakenly made as if bone fragility is present when BMD is ≤−2.5 SD and absent when BMD is >−2.5 SD. This is reflected in the frequent interchangeable description of fractures as “osteoporotic” or “fragility” fractures.^43^, ^44^, ^45^ Finding osteopenia or so‐called “normal” BMD is often a disincentive to initiating treatment because fragility is mistakenly believed to be absent. Even in the setting of a prevalent fracture, treatment may be withheld because the fracture is mistakenly interpreted as being traumatic because of the absence of a diagnosis of “osteoporosis,” particularly when so‐called “normal” BMD is reported.^46^

Second, finding osteoporosis, a BMD *T*‐score ≤ −2.5 SD, is no assurance that fracture will occur. Indeed, only 14.9% and 22.8% of women with osteoporosis had a fracture of any type during 2 and 4 years' follow‐up and only 12.3% and 19.3% had a major fragility fracture during these respective follow‐up times. It was only after 8 years that a substantial number (36.8%) of these women had a fracture of any type and 26.3% had a major fragility fracture. As in women with osteopenia/normal BMD, the SFS detected 76.5%, 76.9%, and 92.9% of the women with osteoporosis having a fracture of any type during 2, 4, and 8 years, respectively, and 71.4%, 72.3%, and 100% of women having a major fragility fracture during these follow‐up times. These findings suggest that the SFS could also be used to identify women with osteoporosis at imminent risk of fracture within 1 to 2 years needing prompt therapy, perhaps initially using an anabolic agent given there is now evidence of superior efficacy over antiresorptives.^47^, ^48^

Third, as mentioned throughout this article, the term so‐called “normal” BMD (*T*‐score > −1.0 SD) in postmenopausal women is a misnomer because it gives the impression that bone strength is normal. Postmenopausal women have lost bone and have microstructural deterioration. At 2 years, of the 97 women having a fracture, 12 had “normal” BMD, similar to the number of women with osteoporosis (*n* = 17). The corresponding numbers of women with normal BMD versus osteoporosis having any type of fracture were 19 and 26 (during 4 years) and 25 and 14 (during 8 years). Of women with “normal” BMD, the SFS ≥70 detected 3/12 (25%) at 2 years, 5/19 (26.3%) at 4 years, and 4/25 (16%) at 8 years. Thus, ironically, the diagnostic threshold of –2.5 SD may result in the failure to treat the very women contributing most of the burden of fractures.

This work has several limitations. There were only 38 women with osteoporosis followed for 8 years. This might account for the lack of association between SFS and incidence of any type of fracture (OR = 3.33, *p* = 0.38) and SFS and major fragility fracture (OR = 3.42, *p* = 0.17). When sample sizes were adequate at 8 years, as in the 347 women with osteopenia, the SFS was predictive (OR = 2.45, *p* = 0.002 for women having any type of fracture; OR = 2.79, *p* = 0.002 for major fragility fracture) with significant AUCs of ~ 0.60 (Supplemental Table S8). Microstructural deterioration in postmenopausal women is likely to be due to bone loss, but a contribution of lower peak values cannot be excluded. Not all women having incident fractures were identified using the SFS, perhaps because abnormalities in material composition are not included in the SFS. Some women identified as being at risk did not have a fracture; a false‐positive rate that may be due to the endpoint of fracture usually requiring a fall as well as severe microstructural deterioration captured by the SFS. We chose the Youden method^26^ to define the SFS threshold. A potential limitation of this method is that it maximizes the sum of the sensitivity and specificity as we regard both as being important, but therefore it does not favor sensitivity over specificity or vice versa. HR‐pQCT is not widely available because of its cost. However, a new smaller commercial HR‐pQCT device, which is FDA cleared and CE marked (conforming with health, safety, and environmental protection standards within European Economic Area), is becoming available for routine clinical use in hospitals, imaging centers, and for primary care physicians to improve access to patients (at similar costs to DXA) that provides the SFS with under three microsievert radiation exposure. Older widely available CT scanners also quantify microstructural deterioration that predicts bone strength^11^ and correlates with measurements using HR‐pQCT (*r* = 0.98), albeit at high radiation exposure.^49^ The findings in these cohorts may not apply to all populations.

In conclusion, microstructural deterioration independently contributes to bone fragility and signals increased fracture risk irrespective of the BMD category. Including a measurement of microstructural deterioration complements the used of BMD by identifying women at imminent, intermediate, and long‐term risk for fragility fracture who otherwise remain undetected by measurement of BMD alone. Treatment slows microstructural deterioration and reduces fracture risk within 6 to 12 months in women with osteopenia,^50^ as well as women with osteoporosis.^51^ Targeting treatment to women ≥70 years at imminent risk for fracture due to microstructural deterioration is likely to curtail the morbidity, mortality, and economic burden of fractures with favorable results. A modeled economic evaluation suggested that screening of women older than 70 years with osteopenia with SFS would be cost‐effective.

## Disclosures

ES has received research support and/or lecture fees from Amgen, Allergan, and Eli Lilly. He has shares in StraxCorp and is a consultant and director on the board of StraxCorp. RZ has shares in StraxCorp and is a director on the board of StraxCorp. All other authors state that they have no conflicts of interest.

## Supporting information


**Supplemental Table S1.** Sample Size and Percentage by Fracture Status for All Data and Separately for the Two Cohorts for Fracture of Any Type
**Supplemental Table S2.** Sample Size and Percentage by Fracture Status for All Data and Separately for the Two Cohorts for Major Fragility Fractures
**Supplemental Table S3.** Association Between Fracture and SFS, BMD, and FRAX, Separately for QUALYOR and OFLEY Cohorts, and the Comparison Between Two Cohorts for All Subjects (p^1^), Women of Any Age With Osteopenia/Normal BMD (p^2^) or Osteoporosis (p^3^) and Women ≥70 Years of Age With Osteopenia/Normal BMD (p^4^)
**Supplemental Table S4.** Association Between Fracture and Composite Score of BMD and SFS for Women of Any Age
**Supplemental Table S5.** Sample Size and Percentage of Fracture and Nonfracture Capture by FRAX, BMD, and SFS for Women of Any Age
**Supplemental Table S6.** Proportion of Fracture Captured by FRAX, BMD, and SFS for Women of Any Age and Women Aged 70 Years and Older
**Supplemental Table S7.** Sample Size and Percentage by Fracture Status for (A) the Women of Any Age With Osteopenia or Normal BMD, (B) Women of Any Age With Osteoporosis, and (C) Women ≥70 Years of Age With Osteopenia or Normal BMD, Having Any Fracture or a Major Fragility Fracture During 2, 4, and 8 Years
**Supplemental Table S8.** ROC Analysis of Any Type of Fracture
**Supplemental Table S9.** ROC Analysis of Major Fragility Fractures
**Supplemental Table S10.** Association Between Incident Fractures and Total Volumetric Bone Mineral Density (vBMD) and Structural Fragility Score (SFS) in Women of Any Age and Women ≥70 Years Showing the Odds Ratio (OR) and 95% Confidence Intervals (CI), *p* Value, Sensitivity, and Specificity
**Supplemental Table S11.** Association Between Fracture and Binary Predictors Trabecular Density, Cortical Porosity, and the Structural Fragility Score (SFS) for Women of any Age
**Supplemental Table S12.** Association Between Fracture and Binary Predictors Trabecular Density, Cortical Porosity, and the Structural Fragility Score (SFS) for Women ≥70 Years
