# Supplementary material for: Deterioration of Cortical and Trabecular Microstructure Identifies Women With Osteopenia or Normal Bone Mineral Density at Imminent and Long‐Term Risk for Fragility Fracture: A Prospective Study
Source: J Bone Miner Res. 2019 Dec 10;35(5):833–44. doi: 10.1002/jbmr.3924 (PMC9328422; doi:10.1002/jbmr.3924)
Supplement: Supplementary file 1 — Supplemental Table S1. Sample Size and Percentage by Fracture Status for All Data and Separately for the Two Cohorts for Fracture of Any Type Supplemental Table S2. Sample Size and Percentage by Fracture Status for All Data and Separately for the Two Cohorts for Major Fragility Fractures Supplemental Table S3. Association Between Fracture and SFS, BMD, and FRAX, Separately for QUALYOR and OFLEY Cohorts, and the Comparison Between Two Cohorts for All Subjects (p1), Women of Any Age With Osteopenia/Normal BMD (p2) or Osteoporosis (p3) and Women ≥70 Years of Age With Osteopenia/Normal BMD (p4) Supplemental Table S4. Association Between Fracture and Composite Score of BMD and SFS for Women of Any Age Supplemental Table S5. Sample Size and Percentage of Fracture and Nonfracture Capture by FRAX, BMD, and SFS for Women of Any Age Supplemental Table S6. Proportion of Fracture Captured by FRAX, BMD, and SFS for Women of Any Age and Women Aged 70 Years and Older Supplemental Table S7. Sample Size and Percentage by Fracture Status for (A) the Women of Any Age With Osteopenia or Normal BMD, (B) Women of Any Age With Osteoporosis, and (C) Women ≥70 Years of Age With Osteopenia or Normal BMD, Having Any Fracture or a Major Fragility Fracture During 2, 4, and 8 Years Supplemental Table S8. ROC Analysis of Any Type of Fracture Supplemental Table S9. ROC Analysis of Major Fragility Fractures Supplemental Table S10. Association Between Incident Fractures and Total Volumetric Bone Mineral Density (vBMD) and Structural Fragility Score (SFS) in Women of Any Age and Women ≥70 Years Showing the Odds Ratio (OR) and 95% Confidence Intervals (CI), p Value, Sensitivity, and Specificity Supplemental Table S11. Association Between Fracture and Binary Predictors Trabecular Density, Cortical Porosity, and the Structural Fragility Score (SFS) for Women of any Age Supplemental Table S12. Association Between Fracture and Binary Predictors Trabecular Density, Cortical Porosity, and the Structural Fra [file JBMR-35-833-s001.docx]

**Table S1** Sample size and percentage by fracture status for all data and separately for the two cohorts for fracture of Any Type.

|  |  | All data | | | | |  | QUALYOR | | | |  |  | OFELY |  |  |
| --- | --- | --- | --- | --- | --- | --- | --- | --- | --- | --- | --- | --- | --- | --- | --- | --- |
|  |  | Fracture | | Non-fracture | | |  | Fracture | | Non-fracture | |  | Fracture | | Non-fracture | |
| Follow-up |  | N | % | | N | % |  | N | % | N | % |  | N | % | N | % |
| 2 years | Total | 97 | 4.62 | | 2003 | 95.4 |  | 66 | 4.29 | 1473 | 95.7 |  | 31 | 5.53 | 530 | 94.5 |
| 4 years | Total | 183 | 8.71 | | 1917 | 91.3 |  | 126 | 8.19 | 1413 | 91.8 |  | 57 | 10.2 | 504 | 89.8 |
|  |  |  |  | |  |  |  |  |  |  |  |  |  |  |  |  |
| 2 years | SFS < 70 | 54 | 55.7 | | 1640 | 81.9 |  | 41 | 62.1 | 1234 | 84.4 |  | 13 | 41.9 | 397 | 74.9 |
|  | ≥ 70 | 43 | 44.3 | | 363 | 18.1 |  | 25 | 37.9 | 230 | 15.6 |  | 18 | 58.1 | 133 | 25.1 |
|  |  |  |  | |  |  |  |  |  |  |  |  |  |  |  |  |
| 4 years | SFS < 70 | 113 | 61.8 | | 1581 | 82.5 |  | 87 | 69.1 | 1197 | 84.7 |  | 26 | 45.6 | 384 | 76.2 |
|  | ≥ 70 | 70 | 38.3 | | 336 | 17.5 |  | 39 | 30.9 | 216 | 15.3 |  | 31 | 54.4 | 120 | 23.8 |
|  |  |  |  | |  |  |  |  |  |  |  |  |  |  |  |  |
| 2 years | BMD > - 2.5 | 80 | 82.5 | | 1906 | 95.2 |  | 56 | 84.9 | 1407 | 95.5 |  | 24 | 77.4 | 499 | 95.4 |
|  | ≤ -2.5 | 17 | 17.5 | | 97 | 4.84 |  | 10 | 15.2 | 66 | 4.48 |  | 7 | 22.6 | 31 | 5.85 |
|  |  |  |  | |  |  |  |  |  |  |  |  |  |  |  |  |
| 4 years | BMD > - 2.5 | 157 | 85.8 | | 1829 | 95.4 |  | 112 | 88.9 | 1351 | 95.6 |  | 45 | 78.9 | 478 | 94.8 |
|  | ≤ -2.5 | 26 | 14.2 | | 88 | 4.59 |  | 14 | 11.1 | 62 | 4.39 |  | 12 | 21.1 | 26 | 5.16 |
|  |  |  |  | |  |  |  |  |  |  |  |  |  |  |  |  |
| 2 years | FRAX ≤ 20 | 91 | 93.8 | | 1959 | 97.8 |  | 64 | 97.0 | 1456 | 98.9 |  | 27 | 87.1 | 503 | 94.9 |
|  | > 20 | 6 | 6.19 | | 44 | 2.20 |  | 2 | 3.00 | 17 | 1.15 |  | 4 | 12.9 | 27 | 5.09 |
|  |  |  |  | |  |  |  |  |  |  |  |  |  |  |  |  |
| 4 years | FRAX ≤ 20 | 171 | 93.4 | | 1879 | 98.0 |  | 122 | 96.8 | 1398 | 98.9 |  | 49 | 86.0 | 481 | 95.4 |
|  | > 20 | 12 | 6.56 | | 38 | 1.98 |  | 4 | 3.17 | 15 | 1.10 |  | 8 | 14.0 | 23 | 4.56 |

Structural Fragility Score (SFS), Fracture Risk Assessment (FRAX) score, Bone Mineral Density (BMD)

**Table S2** Sample size and percentage by fracture status for all data and separately for the two cohorts for Major Fragility Fractures.

|  |  | All data | | | | |  | QUALYOR | | | |  |  | OFLEY |  |  |
| --- | --- | --- | --- | --- | --- | --- | --- | --- | --- | --- | --- | --- | --- | --- | --- | --- |
|  |  | Fracture | | Non-fracture | | |  | Fracture | | Non-fracture | |  | Fracture | | Non-fracture | |
| Follow-up |  | N | % | | N | % |  | N | % | N | % |  | N | % | N | % |
| 2 years | Total | 53 | 2.52 | | 2047 | 97.5 |  | 31 | 2.01 | 1508 | 98.0 |  | 22 | 3.92 | 539 | 96.1 |
| 4 years | Total | 98 | 4.67 | | 2002 | 95.3 |  | 61 | 3.96 | 1478 | 96.0 |  | 37 | 6.60 | 524 | 93.4 |
|  |  |  |  | |  |  |  |  |  |  |  |  |  |  |  |  |
| 2 years | SFS < 70 | 23 | 43.4 | | 1671 | 81.6 |  | 16 | 51.6 | 1268 | 84.1 |  | 7 | 31.8 | 403 | 74.8 |
|  | ≥ 70 | 30 | 56.6 | | 376 | 18.4 |  | 15 | 48.4 | 240 | 15.9 |  | 15 | 68.2 | 136 | 25.2 |
|  |  |  |  | |  |  |  |  |  |  |  |  |  |  |  |  |
| 4 years | SFS < 70 | 49 | 50.0 | | 1645 | 82.2 |  | 37 | 60.7 | 1247 | 84.4 |  | 12 | 32.4 | 398 | 76.0 |
|  | ≥ 70 | 49 | 50.0 | | 357 | 17.8 |  | 24 | 39.3 | 231 | 15.6 |  | 25 | 67.6 | 126 | 24.0 |
|  |  |  |  | |  |  |  |  |  |  |  |  |  |  |  |  |
| 2 years | BMD > - 2.5 | 39 | 73.6 | | 1947 | 95.1 |  | 22 | 71.0 | 1441 | 95.6 |  | 17 | 77.3 | 506 | 93.9 |
|  | ≤ -2.5 | 14 | 26.4 | | 100 | 4.89 |  | 9 | 29.0 | 67 | 4.40 |  | 5 | 22.7 | 33 | 6.12 |
|  |  |  |  | |  |  |  |  |  |  |  |  |  |  |  |  |
| 4 years | BMD > - 2.5 | 76 | 77.6 | | 1910 | 95.4 |  | 48 | 78.7 | 1415 | 95.7 |  | 28 | 75.7 | 495 | 94.5 |
|  | ≤ -2.5 | 22 | 22.5 | | 92 | 4.60 |  | 13 | 21.3 | 63 | 4.26 |  | 9 | 24.3 | 29 | 5.53 |
|  |  |  |  | |  |  |  |  |  |  |  |  |  |  |  |  |
| 2 years | FRAX ≤ 20 | 50 | 94.3 | | 2000 | 97.7 |  | 30 | 96.8 | 1490 | 98.8 |  | 20 | 90.9 | 510 | 94.6 |
|  | > 20 | 3 | 5.66 | | 47 | 2.30 |  | 1 | 3.23 | 18 | 1.19 |  | 2 | 9.10 | 29 | 5.38 |
|  |  |  |  | |  |  |  |  |  |  |  |  |  |  |  |  |
| 4 years | FRAX ≤ 20 | 91 | 92.9 | | 1959 | 97.9 |  | 60 | 98.4 | 1460 | 98.8 |  | 31 | 83.8 | 499 | 95.2 |
|  | > 20 | 7 | 7.14 | | 43 | 2.15 |  | 1 | 1.64 | 18 | 1.22 |  | 6 | 16.2 | 25 | 4.77 |

Structural Fragility Score (SFS), Fracture Risk Assessment (FRAX) score, Bone Mineral Density (BMD)

**Table S3** Association between fracture and SFS, BMD and FRAX, separately for QUALYOR and OFLEY cohorts, and the comparison between two cohorts for all subjects (p^1^), women of any age with osteopenia/normal BMD (p^2^) or osteoporosis (p^3^) and women ≥ 70 years of age with osteopenia/normal BMD (p^4^).

|  |  | QUALYOR | | |  | | OFELY | | |  | |  |  |  |
| --- | --- | --- | --- | --- | --- | --- | --- | --- | --- | --- | --- | --- | --- | --- |
| Follow-up | Variable | OR | 95% CI |  | | OR | | 95% CI |  | | p^1^ | p^2^ | p^3^ | p^4^ |
| Any fracture | | | | | | | | | | | |  |  |  |
| 2 years | SFS | 3.30 | 1.88-5.67 |  | | 4.13 | | 1.85-9.41 |  | | 0.622 | 0.929 | 0.480 | 0.176 |
|  | BMD | 3.81 | 1.65-7.96 |  | | 4.69 | | 1.58-12.3 |  | | 0.724 |  |  |  |
|  | FRAX | 2.68 | 0.29-11.7 |  | | 2.76 | | 0.65-8.75 |  | | 0.974 | 0.936 | 0.281 | 0.817 |
|  |  |  |  |  | |  | |  |  | |  |  |  |  |
| 4 years | SFS | 2.48 | 1.61-3.78 |  | | 3.82 | | 2.10-6.96 |  | | 0.223 | 0.585 | 0.301 | 0.672 |
|  | BMD | 2.72 | 1.36-5.11 |  | | 4.90 | | 2.10-10.9 |  | | 0.233 |  |  |  |
|  | FRAX | 3.06 | 0.73-9.78 |  | | 3.41 | | 1.25-8.42 |  | | 0.877 | 0.976 | 0.480 | 0.852 |
| Major fracture | | | | | | | | | | | |  |  |  |
| 2 years | SFS | 4.95 | 2.24-10.8 |  | | 6.35 | | 2.37-18.7 |  | | 0.674 | 0.866 | 0.520 | 0.175 |
|  | BMD | 8.80 | 3.42-20.8 |  | | 4.51 | | 1.22-13.7 |  | | 0.323 |  |  |  |
|  | FRAX | 2.76 | 0.06-18.6 |  | | 1.76 | | 0.19-7.83 |  | | 0.726 | 0.470 | 0.083 | 0.530 |
|  |  |  |  |  | |  | |  |  | |  |  |  |  |
| 4 years | SFS | 3.50 | 1.96-6.14 |  | | 6.58 | | 3.07-14.8 |  | | 0.164 | 0.332 | 0.355 | 0.689 |
|  | BMD | 6.08 | 2.87-12.1 |  | | 5.49 | | 2.07-13.3 |  | | 0.850 |  |  |  |
|  | FRAX | 1.35 | 0.03-8.85 |  | | 3.86 | | 1.20-10.6 |  | | 0.355 | 0.129 | 0.228 | 0.168 |

Structural Fragility Score (SFS), Fracture Risk Assessment (FRAX) score, Bone Mineral Density (BMD). Confidence intervals (CI) for odds ratios (OR) were computed using the exact method and these statistics were presented for the whole data set only; Mantel-Haenszel (M-H) method was used to compute p-value (p) to compare ORs between QUALYOR and OFELY. Only OFELY has 8 years follow-up so comparison is not possible.

**Table S4** Association between fracture and composite score of BMD and SFS for women of any age.

|  |  | Any Type of Fractures | | |  | | Major Fragility Fractures | | |
| --- | --- | --- | --- | --- | --- | --- | --- | --- | --- |
| Follow-up | Predictor | OR | 95% CI | p |  | OR | | 95% CI | p |
| 2 years | SFS < 70 BMD > -2.5 SD | *Ref |  |  |  | *Ref | |  |  |
|  | SFS < 70 BMD ≤ -2.5 SD | 2.44 | (0.85, 7.02) | 0.097 |  | 6.55 | | (2.15, 19.9) | 0.001 |
|  | SFS ≥ 70 BMD > -2.5 SD | 3.00 | (1.88, 4.79) | 4.4x10^-6^ |  | 5.20 | | (2.74, 9.84) | 4.4x10^-7^ |
|  | SFS ≥ 70 BMD ≤ -2.5 SD | 9.18 | (4.66, 18.1) | 1.6x10^-10^ |  | 17.8 | | (7.84, 40.2) | 5.6x10^-12^ |
| 4 years | SFS < 70 BMD > -2.5 SD | Ref |  |  |  | Ref | |  |  |
|  | SFS < 70 BMD ≤ -2.5 SD | 1.72 | (0.72, 4.10) | 0.223 |  | 4.45 | | (1.81, 10.9) | 0.001 |
|  | SFS ≥ 70 BMD > -2.5 SD | 2.40 | (1.68, 3.43) | 1.6x10^-6^ |  | 3.89 | | (2.43, 6.21) | 1.4x10^-8^ |
|  | SFS ≥ 70 BMD ≤ -2.5 SD | 7.53 | (4.23, 13.4) | 6.4x10^-12^ |  | 14.1 | | (7.37, 27.1) | 1.6x10^-15^ |
| 8 years | SFS < 70 BMD > -2.5 SD | Ref |  |  |  | Ref | |  |  |
|  | SFS < 70 BMD ≤ -2.5 SD | 1.24 | (0.14, 10.8) | 0.844 |  | 0.93 | | (0.01, 6.55) | 0.999 |
|  | SFS ≥ 70 BMD > -2.5 SD | 2.70 | (1.66, 4.37) | 5.6x10^-5^ |  | 3.62 | | (2.03, 6.43) | 1.2x10^-5^ |
|  | SFS ≥ 70 BMD ≤ -2.5 SD | 4.25 | (1.99, 9.09) | 0.0002 |  | 5.88 | | (2.54, 13.6) | 3.5x10^-5^ |

*Referent = Reference category; SFS = Structural Fragility Score; BMD = Bone Mineral Density.

The Odds Ratios (OR) with the 95% confidence intervals (CI) shown graphically in figure 3 are provided here with p values. Reduced BMD ≤ -2.5 SD is not predictive unless SFS is high (≥70) and BMD > -2.5 SD, i.e., in the osteopenic or normal range, is associated with fracture when SFS ≥ 70, i.e., if there is microstructural deterioration.

**Table S5** Sample size and percentage of fracture and non-fracture capture by FRAX, BMD and SFS for women of any age.

|  | Fracture of Any Type | | | |  | Major Fragility Fracture | | | | |
| --- | --- | --- | --- | --- | --- | --- | --- | --- | --- | --- |
|  | Fracture | | Non-fracture | |  | | Fracture | | Non-fracture | |
| Variable | N | % | N | % |  | | N | % | N | % |
| FRAX ≤ 20 | 97 | 91.5 | 433 | 95.2 |  | | 58 | 89.2 | 472 | 95.2 |
| >20 | 9 | 8.49 | 22 | 4.84 |  | | 7 | 10.8 | 24 | 4.84 |
| BMD > - 2.5 | 92 | 86.8 | 431 | 94.7 |  | | 55 | 84.6 | 468 | 94.4 |
| ≤ -2.5 | 14 | 13.2 | 24 | 5.27 |  | | 10 | 15.4 | 28 | 5.65 |
| SFS < 70 | 57 | 53.8 | 353 | 77.6 |  | | 29 | 44.6 | 381 | 76.8 |
| ≥ 70 | 49 | 46.2 | 102 | 22.4 |  | | 36 | 55.4 | 115 | 23.2 |

Structural Fragility Score (SFS), Fracture Risk Assessment (FRAX) score, Bone Mineral Density (BMD).

**Table S6** Proportion of fracture captured by FRAX, BMD and SFS for women of any age and women aged 70 years and older.

|  |  | FRAX | BMD | SFS |  |  |  |
| --- | --- | --- | --- | --- | --- | --- | --- |
|  | N | n (%) | n (%) | n (%) | p^1^ | p^2^ | p^3^ |
| Women of any age Any Type of Fracture | | | | | | | |
| 2 years | 97 | 6 (6.19) | 17 (17.5) | 43 (44.3) | 0.0148 | <0.0001 | <0.0001 |
| 4 years | 183 | 12 (6.56) | 26 (14.2) | 70 (38.3) | 0.0166 | <0.0001 | <0.0001 |
| 8 years | 106 | 9 (8.49) | 14 (13.2) | 49 (46.2) | 0.2701 | <0.0001 | <0.0001 |
| Major Fragility Fractures | | | | | | | |
| 2 years | 53 | 3 (5.66) | 14 (26.4) | 30 (56.6) | 0.0036 | <0.0001 | 0.0016 |
| 4 years | 98 | 7 (7.14) | 22 (22.5) | 49 (50.0) | 0.0025 | <0.0001 | 0.0001 |
| 8 years | 65 | 7 (10.8) | 10 (15.4) | 36 (55.4) | 0.4370 | <0.0001 | <0.0001 |
| Women aged 70 years and older Any Type of Fracture | | | | | | | |
| 2 years | 38 | 6 (15.8) | 10 (26.3) | 26 (68.4) | 0.2616 | <0.0001 | 0.0002 |
| 4 years | 73 | 12 (16.4) | 16 (21.9) | 45 (61.6) | 0.3984 | <0.0001 | <0.0001 |
| 8 years | 55 | 9 (16.4) | 12 (21.8) | 40 (72.7) | 0.4713 | <0.0001 | <0.0001 |
| Major Fragility Fractures | | | | | | | |
| 2 years | 24 | 3 (12.5) | 7 (29.2) | 19 (79.2) | 0.1544 | <0.0001 | 0.0005 |
| 4 years | 48 | 7 (14.6) | 12 (25.0) | 35 (72.9) | 0.2011 | <0.0001 | <0.0001 |
| 8 years | 40 | 7 (17.5) | 9 (22.5) | 31 (77.5) | 0.5762 | <0.0001 | <0.0001 |

Structural Fragility Score (SFS), Fracture Risk Assessment (FRAX) score, Bone Mineral Density (BMD). N = number of fractures; n = number of fractures capture by FRAX, BMD and SFS. P-value (p) compares the difference in proportions between ^1^FRAX versus BMD, ^2^FRAX versus SFS and ^3^BMD versus SFS; all p-values (p) <0.05 remained <0.05 after adjusting for multiple testing using Bonferroni correction.

**Table S7** Sample size and percentage by fracture status for (A) the women of any age with osteopenia or normal BMD, (B) women of any age with osteoporosis and (C) women ≥ 70 years of age with osteopenia or normal BMD, having Any Fracture or a Major Fragility Fracture during 2, 4 and 8 years.

|  | |  | | Any Fracture | | | | | | |  | | Major fracture | | | | | | | | | |
| --- | --- | --- | --- | --- | --- | --- | --- | --- | --- | --- | --- | --- | --- | --- | --- | --- | --- | --- | --- | --- | --- | --- |
|  | |  | | Fracture | | | | | Non-fracture | |  | | Fracture | | | | | Non-fracture | | | | |
| Follow-up | | Variable | | N | | % | | N | | % |  | | N | | % | | N | | | % | | |
| (A) Women of any age with osteopenia or normal BMD | | | | | | | | | | | | | | | | | | | | | | |
| 2 years | FRAX ≤20 | | 78 | | 97.5 | | 1875 | | | 98.4 | |  | 38 | 97.4 | | 1915 | | | | | 98.4 | |
|  | >20 | | 2 | | 2.50 | | 31 | | | 1.63 | |  | 1 | 2.56 | | 32 | | | | | 1.64 | |
|  | SFS <70 | | 50 | | 62.5 | | 1588 | | | 83.3 | |  | 19 | 48.7 | | 1619 | | | | | 83.2 | |
|  | ≥70 | | 30 | | 37.5 | | 318 | | | 16.7 | |  | 20 | 51.3 | | 328 | | | | | 16.9 | |
| 4 years | FRAX ≤20 | | 151 | | 96.2 | | 1802 | | | 98.5 | |  | 73 | 96.1 | | 1880 | | | | | 98.4 | |
|  | >20 | | 6 | | 3.82 | | 27 | | | 1.48 | |  | 3 | 3.95 | | 30 | | | | | 1.57 | |
|  | SFS <70 | | 107 | | 68.2 | | 1531 | | | 83.7 | |  | 43 | 56.6 | | 1595 | | | | | 83.5 | |
|  | ≥70 | | 50 | | 31.9 | | 298 | | | 16.3 | |  | 33 | 43.4 | | 315 | | | | | 16.7 | |
| 8 years | FRAX ≤20 | | 88 | | 95.7 | | 419 | | | 97.2 | |  | 51 | 92.7 | | 456 | | | | | 97.4 | |
|  | >20 | | 4 | | 4.35 | | 12 | | | 2.78 | |  | 4 | 7.27 | | 12 | | | | | 2.56 | |
|  | SFS <70 | | 56 | | 60.9 | | 348 | | | 80.7 | |  | 29 | 52.7 | | 375 | | | | | 80.1 | |
|  | ≥70 | | 36 | | 39.1 | | 83 | | | 19.3 | |  | 26 | 47.3 | | 93 | | | | | 19.9 | |
| (B) Women of any age with osteoporosis | | | | | | | | | | | | | | | | | | | | | | |
| 2 years | FRAX ≤20 | | 13 | | 76.5 | | 84 | | | 86.6 |  | | 12 | 85.7 | | 85 | | | 85.0 | | |  |
|  | >20 | | 4 | | 23.5 | | 13 | | | 13.4 |  | | 2 | 14.3 | | 15 | | | 15.0 | | |  |
|  | SFS <70 | | 4 | | 23.5 | | 52 | | | 53.6 |  | | 4 | 28.6 | | 52 | | | 52.0 | | |  |
|  | ≥70 | | 13 | | 76.5 | | 45 | | | 46.4 |  | | 10 | 71.4 | | 48 | | | 48.0 | | |  |
| 4 years | FRAX ≤20 | | 20 | | 76.9 | | 77 | | | 87.5 |  | | 18 | 81.8 | | 79 | | | 85.9 | | |  |
|  | >20 | | 6 | | 23.1 | | 11 | | | 12.5 |  | | 4 | 18.2 | | 13 | | | 14.1 | | |  |
|  | SFS <70 | | 6 | | 23.1 | | 50 | | | 56.8 |  | | 6 | 27.3 | | 50 | | | 54.4 | | |  |
|  | ≥70 | | 20 | | 76.9 | | 38 | | | 43.2 |  | | 16 | 72.7 | | 42 | | | 45.7 | | |  |
| 8 years | FRAX ≤20 | | 9 | | 64.3 | | 14 | | | 58.3 |  | | 7 | 70.0 | | 16 | | | 57.1 | | |  |
|  | >20 | | 5 | | 35.7 | | 10 | | | 41.7 |  | | 3 | 30.0 | | 12 | | | 42.9 | | |  |
|  | SFS <70 | | 1 | | 7.14 | | 5 | | | 20.8 |  | | 0 | 0.00 | | 6 | | | 21.4 | | |  |
|  | ≥70 | | 13 | | 92.9 | | 19 | | | 79.2 |  | | 10 | 100 | | 22 | | | 78.6 | | |  |
| (C) Women 70 and over with osteopenia or normal BMD | | | | | | | | | | | | | | | | | | | | | | |
| 2 years | FRAX ≤20 | | 26 | | 92.9 | | 510 | | | 94.8 |  | | 16 | 94.1 | | 520 | | | 94.7 | | |  |
|  | >20 | | 2 | | 7.14 | | 28 | | | 5.20 |  | | 1 | 5.88 | | 29 | | | 5.28 | | |  |
|  | SFS <70 | | 10 | | 35.7 | | 391 | | | 72.7 |  | | 3 | 17.7 | | 398 | | | 72.5 | | |  |
|  | ≥70 | | 18 | | 64.3 | | 147 | | | 27.3 |  | | 14 | 82.3 | | 151 | | | 27.5 | | |  |
| 4 years | FRAX ≤20 | | 51 | | 89.5 | | 485 | | | 95.3 |  | | 33 | 91.7 | | 503 | | | 94.9 | | |  |
|  | >20 | | 6 | | 10.5 | | 24 | | | 4.72 |  | | 3 | 8.33 | | 27 | | | 5.09 | | |  |
|  | SFS <70 | | 26 | | 45.6 | | 375 | | | 73.7 |  | | 11 | 30.6 | | 390 | | | 73.6 | | |  |
|  | ≥70 | | 31 | | 54.4 | | 134 | | | 26.3 |  | | 25 | 69.4 | | 140 | | | 26.4 | | |  |
| 8 years | FRAX ≤20 | | 39 | | 90.7 | | 133 | | | 93.0 |  | | 27 | 87.1 | | 145 | | | 93.6 | | |  |
|  | >20 | | 4 | | 9.30 | | 10 | | | 6.99 |  | | 4 | 12.9 | | 10 | | | 6.45 | | |  |
|  | SFS <70 | | 15 | | 34.9 | | 98 | | | 68.5 |  | | 9 | 29.0 | | 104 | | | 67.1 | | |  |
|  | ≥70 | | 28 | | 65.1 | | 45 | | | 31.5 |  | | 22 | 71.0 | | 51 | | | 32.9 | | |  |

Structural Fragility Score (SFS), Fracture Risk Assessment (FRAX) score, Bone Mineral Density (BMD).

**Table S8** ROC analysis of Any Type of Fracture.

|  | 2 years | | |  | | 4 years | |  | | | 8 years | |
| --- | --- | --- | --- | --- | --- | --- | --- | --- | --- | --- | --- | --- |
|  | AUC | | 95% CI |  | AUC | | 95% CI | |  | AUC | | 95% CI |
| All data |  | |  |  |  | |  | |  |  | |  |
| SFS | 0.630 | | 0.57-0.69 |  | 0.618 | | 0.57-0.66 | |  | 0.614 | | 0.55-0.67 |
| BMD | 0.555 | | 0.49-0.69 |  | 0.581 | | 0.54-0.62 | |  | 0.614 | | 0.55-0.67 |
| FRAX | 0.556 | | 0.50-0.61 |  | 0.564 | | 0.52-0.60 | |  | 0.541 | | 0.48-0.60 |
| Women of any age with osteopenia or normal BMD | | | | | | | | |  |  | |  |
| SFS | | 0.601 | 0.54-0.67 |  | 0.592 | | 0.54-0.64 | |  | 0.605 | | 0.54-0.67 |
| BMD | | 0.489 | 0.43-0.55 |  | 0.540 | | 0.50-0.58 | |  | 0.587 | | 0.53-0.65 |
| FRAX | | 0.517 | 0.46-0.58 |  | 0.541 | | 0.50-0.58 | |  | 0.524 | | 0.46-0.59 |
| Women of any age with osteoporosis | | | | | | |  | |  |  | |  |
| SFS | | 0.637 | 0.51-0.76 |  | 0.670 | | 0.56-0.78 | |  | 0.458 | | 0.28-0.64 |
| BMD | | 0.561 | 0.41-0.71 |  | 0.576 | | 0.45-0.70 | |  | 0.531 | | 0.35-0.72 |
| FRAX | | 0.574 | 0.43-0.72 |  | 0.578 | | 0.46-0.70 | |  | 0.466 | | 0.28-0.66 |
| Women 70 and over with osteopenia or normal BMD | | | | | | | | |  |  | |  |
| SFS | | 0.651 | 0.52-0.78 |  | 0.635 | | 0.55-0.72 | |  | 0.686 | | 0.58-0.79 |
| BMD | | 0.517 | 0.41-0.62 |  | 0.553 | | 0.48-0.63 | |  | 0.611 | | 0.51-0.71 |
| FRAX | | 0.529 | 0.42-0.64 |  | 0.564 | | 0.49-0.64 | |  | 0.529 | | 0.42-0.63 |

Structural Fragility Score (SFS), Fracture Risk Assessment (FRAX) score, Bone Mineral Density (BMD).

**Table S9** ROC analysis of Major Fragility Fractures.

|  | 2 years | |  | | | | 4 years | | | |  | | | | 8 years | | |  |
| --- | --- | --- | --- | --- | --- | --- | --- | --- | --- | --- | --- | --- | --- | --- | --- | --- | --- | --- |
|  | AUC | 95% CI | |  | | | AUC | 95% CI | |  | | | AUC | | | 95% CI | |  |
| All data |  |  | |  | | |  |  | |  | | |  | | |  | |  |
| SFS | 0.665 | 0.58-0.75 | |  | | | 0.671 | 0.61-0.73 | |  | | | 0.670 | | | 0.60-0.74 | |  |
| BMD | 0.594 | 0.51-0.68 | |  | | | 0.617 | 0.56-0.68 | |  | | | 0.628 | | | 0.56-0.70 | |  |
| FRAX | 0.549 | 0.48-0.62 | |  | | | 0.567 | 0.51-0.62 | |  | | | 0.562 | | | 0.49-0.63 | |  |
| Women of any age with osteopenia or normal BMD | | | | | | | | | |  | | |  | | |  | |  |
| SFS | 0.632 | 0.54-0.73 | |  | | 0.641 | | | 0.57-0.71 | | |  | | 0.655 | | | 0.58-0.73 | |
| BMD | 0.490 | 0.40-0.58 | |  | | 0.541 | | | 0.48-0.61 | | |  | | 0.600 | | | 0.52-0.68 | |
| FRAX | 0.518 | 0.43-0.61 | |  | | 0.541 | | | 0.48-0.60 | | |  | | 0.562 | | | 0.49-0.63 | |
| Women of any age with osteoporosis | | | | | | | |  | | | |  |  | | |  | |  |
| SFS | 0.595 | 0.46-0.73 | |  | 0.635 | | | 0.51-0.76 | | | |  | 0.562 | | | 0.40-0.73 | |  |
| BMD | 0.542 | 0.38-0.70 | |  | 0.573 | | | 0.44-0.70 | | | |  | 0.524 | | | 0.32-0.72 | |  |
| FRAX | 0.494 | 0.34-0.65 | |  | 0.531 | | | 0.40-0.66 | | | |  | 0.424 | | | 0.22-0.63 | |  |
| Women 70 and over with osteopenia or normal BMD | | | | | | | | | | | |  |  | | |  | |  |
| SFS | 0.741 | 0.58-0.91 | |  | 0.715 | | | 0.62-0.81 | | | |  | 0.771 | | | 0.69-0.86 | |  |
| BMD | 0.514 | 0.37-0.66 | |  | 0.542 | | | 0.45-0.64 | | | |  | 0.595 | | | 0.48-0.71 | |  |
| FRAX | 0.519 | 0.37-0.67 | |  | 0.541 | | | 0.44-0.64 | | | |  | 0.576 | | | 0.46-0.69 | |  |

Structural Fragility Score (SFS), Fracture Risk Assessment (FRAX) score, Bone Mineral Density (BMD).

**Table S10**  Association between incident fractures and total volumetric bone mineral density (vBMD) and Structural Fragility Score (SFS) in women of any age and women ≥ 70 years showing the Odds Ratio (OR) and 95% confidence intervals (CI), p value, sensitivity and specificity.

| Any Type of Fracture | | OR (95% CIs) | p | Sensitivity | Specificity |
| --- | --- | --- | --- | --- | --- |
| 2 year ≥ 70 yrs | total vBMD | 2.71 (1.40-5.27) | 0.0030 | 61% | 64% |
|  | SFS | 4.84 (2.41-9.71) | <0.0001 | 68% | 70% |
| Any age | total vBMD | 2.90 (1.92-4.37) | <0.0001 | 46% | 77% |
|  | SFS | 3.68 (2.43-5.58) | <0.0001 | 45% | 82% |
| 4 year ≥ 70 yrs | total vBMD | 2.54 (1.55-4.17) | 0.0002 | 58% | 65% |
|  | SFS | 3.93 (2.37-6.50) | <0.0001 | 62% | 71% |
| Any age | total vBMD | 2.42 (1.77-3.31) | <0.0001 | 41% | 78% |
|  | SFS | 2.96 (2.15-4.08) | <0.0001 | 39% | 83% |
| 8 year ≥ 70 yrs | total vBMD | 2.57 (1.36-4.87) | 0.0040 | 67% | 56% |
|  | SFS | 4.30 (2.21-8.37) | <0.0001 | 73% | 62% |
| Any age | total vBMD | 2.25 (1.46-3.47) | 0.0002 | 47% | 72% |
|  | SFS | 2.97 (1.91-4.61) | <0.0001 | 46% | 78% |
| Major Fragility Fractures | |  | | | |
| 2 year ≥ 70 yrs | total vBMD | 3.42 (1.47-7.96) | 0.0040 | 67% | 64% |
|  | SFS | 7.92 (3.03-20.7) | <0.0001 | 79% | 69% |
| Any age | total vBMD | 4.23 (2.44-7.34) | <0.0001 | 56% | 77% |
|  | SFS | 6.04 (3.46-10.5) | <0.0001 | 58% | 82% |
| 4 year ≥ 70 yrs | total vBMD | 3.67 (1.98-6.81) | <0.0001 | 67% | 65% |
|  | SFS | 6.32 (3.29-12.1) | <0.0001 | 73% | 71% |
| Any age | total vBMD | 3.69 (2.45-5.56) | <0.0001 | 52% | 78% |
|  | SFS | 4.71 (3.12-7.12) | <0.0001 | 51% | 82% |
| 8 year ≥ 70 yrs | total vBMD | 2.39 (1.17-4.89) | 0.0170 | 68% | 54% |
|  | SFS | 5.03 (2.29-11.0) | 0.0001 | 78% | 60% |
| Any age | total vBMD | 2.84 (1.69-4.78) | 0.0001 | 54% | 71% |
|  | SFS | 4.09 (2.41-6.93) | <0.0001 | 55% | 77% |

The sample sizes in this analysis were 2093 (all women) and 610 (women ≥ 70 years). Association analyses were conducted using penalized maximum likelihood logistic regression. The threshold of total vBMD corresponding to SFS 70 was 231 mgHA/cc.

**Table S11**: Association between fracture and binary predictors trabecular density, cortical porosity and the Structural Fragility Score (SFS) for women of any age.

|  | Trabecular Density | | |  | Trabecular Density without Cortical porosity | | |  | Cortical Porosity | | | Cortical Porosity without Trabecular Density | | |  | Trabecular Density and Cortical Porosity | | |  | SFS | | |
| --- | --- | --- | --- | --- | --- | --- | --- | --- | --- | --- | --- | --- | --- | --- | --- | --- | --- | --- | --- | --- | --- | --- |
|  | OR | p | 95% CI |  | OR | p | 95% CI |  | OR | p | 95%CI | OR | p | 95%CI |  | OR | p | 95%CI |  | OR | p | 95%CI |
| Any Type of Fracture | | | | | | | | | | | | | | | | | | | | | | |
| 2yrs | 2.73 | <0.001 | 1.81-4.13 |  | 1.37 | 0.340 | 0.72-2.62 |  | 2.25 | <0.001 | 1.44-3.50 | 1.00 | 0.989 | 0.66-1.52 |  | 3.02 | <0.001 | 1.94-4.69 |  | 3.60 | <0.001 | 2.37-5.46 |
| 4yrs | 2.54 | <0.001 | 1.86-3.47 |  | 1.41 | 0.159 | 0.87-2.29 |  | 1.59 | 0.004 | 1.16-2.17 | 0.79 | 0.159 | 0.57-1.10 |  | 2.76 | <0.001 | 1.96-3.89 |  | 2.91 | <0.001 | 2.12-4.02 |
| 8yrs | 1.91 | 0.004 | 1.23-2.95 |  | 0.56 | 0.197 | 0.23-1.35 |  | 1.89 | 0.005 | 1.21-2.93 | 0.86 | 0.514 | 0.54-1.36 |  | 2.61 | <0.001 | 1.65-4.14 |  | 2.98 | <0.001 | 1.91-4.62 |
| Major Fragility Fractures | | | | | | | | | | | | | | | | | | | | | | |
| 2yrs | 3.00 | <0.001 | 1.73-5.19 |  | 0.41 | 0.215 | 0.10-1.68 |  | 3.63 | <0.001 | 1.86-7.10 | 0.95 | 0.855 | 0.54-1.67 |  | 4.64 | <0.001 | 2.66-8.11 |  | 5.80 | <0.001 | 3.33-10.1 |
| 4yrs | 2.93 | <0.001 | 1.94-4.42 |  | 0.68 | 0.362 | 0.29-1.57 |  | 2.67 | <0.001 | 1.69-4.22 | 0.86 | 0.479 | 0.56-1.32 |  | 4.17 | <0.001 | 2.73-6.37 |  | 4.61 | <0.001 | 3.05-6.96 |
| 8yrs | 2.47 | 0.001 | 1.46-4.16 |  | 0.30 | 0.098 | 0.07-1.25 |  | 2.71 | 0.001 | 1.51-4.85 | 0.80 | 0.439 | 0.45-1.42 |  | 3.76 | <0.001 | 2.20-6.44 |  | 4.11 | <0.001 | 2.42-7.00 |

Odds ratio (OR). Thresholds are the 90^th^ centile (42.2%) for cortical porosity and 5^th^ centile (4.8 mgHA/cc) for trabecular density, ≥70 units for SFS.

**Table S12** Association between fracture and binary predictors trabecular density, cortical porosity and the Structural Fragility Score (SFS) for women ≥ 70 years.

|  | | | Trabecular Density | | | |  | Trabecular Density without Cortical porosity | | |  | Cortical Porosity | | |  | Cortical Porosity without Trabecular Density | | |  | Trabecular Density and Cortical Porosity | | |  | SFS | | |
| --- | --- | --- | --- | --- | --- | --- | --- | --- | --- | --- | --- | --- | --- | --- | --- | --- | --- | --- | --- | --- | --- | --- | --- | --- | --- | --- |
|  | | | OR | | p | 95% CI |  | OR | p | 95% CI |  | OR | p | 95%CI |  | OR | p | 95%CI |  | OR | p | 95%CI |  | OR | p | 95%CI |
| Any Type of Fracture | | | | | | | | | | | | | | | | | | | | | | | | | | |
| 2yrs | | 2.91 | | 0.002 | | 1.42-6.04 |  | 0.35 | 0.504 | 0.01-2.21 |  | 6.93 | 0.001 | 1.75-60.1 |  | 0.74 | 0.406 | 0.36-1.51 |  | 3.80 | 0.0001 | 1.84-7.86 |  | 4.98 | <0.0001 | 2.36-11.1 |
| 4yrs | | 2.55 | | <0.001 | | 1.51-4.32 |  | 0.55 | 0.460 | 0.11-1.81 |  | 2.47 | 0.007 | 1.21-5.53 |  | 0.65 | 0.105 | 0.38-1.10 |  | 3.18 | <0.0001 | 1.86-5.42 |  | 3.98 | <0.0001 | 2.34-6.89 |
| 8yrs | | 1.90 | | 0.043 | | 0.98-3.74 |  | 0.21 | 0.071 | 0.04-1.14 |  | 2.45 | 0.041 | 1.00-6.93 |  | 0.60 | 0.150 | 0.29-1.20 |  | 2.88 | 0.0011 | 1.47-5.72 |  | 4.37 | <0.0001 | 2.14-9.28 |
| Major Fragility Fractures | | | | | | | | | | | | | | | | | | | | | | | | | | |
| 2yrs | 3.22 | | | 0.006 | | 1.30-8.28 |  | 0.26 | 0.353 | 0.02-4.40 |  | 18.6 | 0.041 | 1.13-308 |  | 0.73 | 0.535 | 0.29-1.80 |  | 4.65 | 0.0003 | 1.87-12.0 |  | 8.53 | <0.0001 | 3.02-29.7 |
| 4yrs | 2.55 | | | <0.001 | | 1.51-4.32 |  | 0.55 | 0.460 | 0.11-1.81 |  | 2.47 | 0.007 | 1.21-5.53 |  | 0.65 | 0.105 | 0.38-1.10 |  | 4.19 | <0.0001 | 2.20-8.05 |  | 6.50 | <0.0001 | 3.26-13.8 |
| 8yrs | 2.56 | | | 0.013 | | 1.19-5.70 |  | 0.22 | 0.212 | 0.01-1.50 |  | 3.13 | 0.036 | 1.03-12.8 |  | 0.51 | 0.106 | 0.22-1.14 |  | 3.58 | 0.0005 | 1.67-7.94 |  | 5.20 | <0.0001 | 2.25-13.2 |

Odds ratio (OR). Thresholds are the 90^th^ centile (42.2%) for cortical porosity and 5^th^ centile (4.8 mgHA/cc) for trabecular density, ≥70 units for SFS.
